# Three‐dimensional facial morphology in Cantú syndrome

**DOI:** 10.1002/ajmg.a.61517

**Published:** 2020-02-26

**Authors:** Helen I. Roessler, Kathleen Shields, Dorothy K. Grange, Nine V.A.M. Knoers, Gijs van Haaften, Peter Hammond, Mieke M. van Haelst

**Affiliations:** ^1^ Department of Genetics, Center for Molecular Medicine University Medical Center Utrecht, Utrecht University Utrecht The Netherlands; ^2^ Division of Genetics and Genomic Medicine, Department of Pediatrics Washington University School of Medicine St. Louis Missouri USA; ^3^ Center for the Investigation of Membrane Excitability Diseases (CIMED) St. Louis Missouri USA; ^4^ Deptartment of Genetics University Medical Center Groningen Groningen The Netherlands; ^5^ Department of Human Genetics KU Leuven Leuven Belgium; ^6^ Department of Clinical Genetics, Amsterdam Medical Center University of Amsterdam Amsterdam The Netherlands; ^7^ Department of Clinical Genetics, VU Medical Center VU University Amsterdam Amsterdam The Netherlands

**Keywords:** 3D imaging, Cantú syndrome, dense surface model (DSM), dysmorphology, facial phenotyping, principal component analysis (PCA)

## Abstract

Cantú syndrome (CS) was first described in 1982, and is caused by pathogenic variants in *ABCC9* and *KCNJ8* encoding regulatory and pore forming subunits of ATP‐sensitive potassium (K_ATP_) channels, respectively. It is characterized by congenital hypertrichosis, osteochondrodysplasia, extensive cardiovascular abnormalities and distinctive facial anomalies including a broad nasal bridge, long philtrum, epicanthal folds, and prominent lips. Many genetic syndromes, such as CS, involve facial anomalies that serve as a significant clue in the initial identification of the respective disorder before clinical or molecular diagnosis are undertaken. However, an overwhelming number of CS patients receive misdiagnoses based on an evaluation of coarse facial features. By analyzing three‐dimensional images of CS faces, we quantified facial dysmorphology in a cohort of both male and female CS patients with confirmed *ABCC9* variants. Morphometric analysis of different regions of the face revealed gender‐specific significant differences in face shape. Moreover, we show that 3D facial photographs can distinguish between CS and other genetic disorders with specific facial dysmorphologies that have been mistaken for CS‐associated anomalies in the past, hence assisting in an earlier clinical and molecular diagnosis. This optimizes genetic counseling and reduces stress for patients and parents by avoiding unnecessary misdiagnosis.

## 1 INTRODUCTION

Cantú syndrome (CS; OMIM #239850) is a rare autosomal dominant condition characterized by a wide constellation of clinical features, namely coarse facial features, congenital hypertrichosis, osteochondrodysplasia and extensive cardiovascular anomalies including cardiomegaly, patent ductus arteriosus (PDA), pericardial effusion and dilated and torturous cerebral blood vessels (Grange et al., 2019; Grange, Lorch, Cole, & Singh, 2006; Leon Guerrero et al., 2016; Scurr et al., 2011). CS is caused by gain‐of‐function (GoF) pathogenic variants in *ABCC9* and, less commonly, in *KCNJ8*, which encode the regulatory (SUR2) and pore‐forming (Kir6.1) subunits, respectively, of ATP‐sensitive potassium (K_ATP_) channels(Brownstein et al., 2013; Cooper et al., 2014; Harakalova et al., 2012; McClenaghan et al., 2017; van Bon et al., 2012). Notably, a majority of cases are considered to harbor de novo variants.

Remarkably, there is considerable variation in the phenotypic spectrum, even within family members sharing the same *ABCC9* variant (Roessler, Volker‐Touw, Terhal, van Haaften, & van Haelst, 2018). The underlying reason for this heterogeneous clinical presentation is still unclear.

Dysmorphic facial features, on the other hand, are observed in every CS patient and are usually evident at birth. In younger patients, features include a low frontal hairline, epicanthal folds, flat nasal bridge, anteverted nares, long philtrum, macroglossia, prominent mouth, and thick lips. Additionally, the palate may be high arched and/or narrow, the gingiva may be thickened and an anterior open bite may be present (Grange et al., 2019). With advancing age, however, these clinical features are subject to considerable phenotypic changes. Whereas the initial facial shape appears round with full cheeks, the facial phenotype progresses to a lengthening of the face with a pointed chin and a more prominent forehead (Scurr et al., 2011). Progressive coarsening of the face results in further flattening of the nasal bridge with prominent supra‐orbital ridges and fuller lips. The small nose becomes more bulbous. Periorbital fullness seems to be consistent (Roessler et al., 2018).

Despite the identification of *ABCC9* and *KCNJ8* pathogenic variants as being causal, an explanation for variability of CS‐associated features remains unknown. The correlation between GoF mutations in K_ATP_ channels and the coarse facial appearance is currently still not understood. Additionally, in a recent report describing 74 patients in the International Cantú Syndrome Registry (ICSR) we were unable to correlate genotype to specific phenotypic features in CS individuals including facial appearance (Grange et al., 2019).

Facial dysmorphism plays an important role in the clinical diagnosis of genetic conditions such as CS since it often presents as a preliminary clue before clinical examination and molecular genotyping are undertaken. Since the genes associated with CS have only recently been identified and not all patients have had adequate genetic counseling and investigation, we suspect that there is still a large number of undiagnosed or misdiagnosed cases. Additionally, the low incidence of CS limits exposure during training of clinical geneticists and inhibits the development of skills in recognizing the facial features characteristic of this syndrome. An overwhelming number of CS patients initially received other diagnoses, often based on coarse facial features with mucopolysacharidosis being most common; an unnecessary and stressful process for parents (Grange et al., 2019). Hence, quantitative analysis of the described facial morphology is of vital importance in characterizing the phenotype, which will assist in achieving earlier clinical and molecular diagnosis and thus optimizing appropriate medical care and genetic counseling.

Three‐dimensional (3D) surface imaging systems have already been successfully applied in a range of clinical situations such as burns (Kovacs et al., 2005), dermatology (Ardehali et al., 2007) and orthodontics (Hajeer, Millett, Ayoub, & Siebert, 2004). Rapid, noninvasive 3D imaging enables the face to be viewed from any angle and at closer proximity than most children, or even adults, would tolerate with a human observer. Consequently, 3D face analysis using dense surface modeling (DSM) has proven successful in delineating facial morphology in Williams syndrome (WS; OMIM # 194050) (Hammond & Suttie, 2012), Noonan syndrome (NS; OMIM #163950) (Hammond, 2007), Wolf–Hirschhorn syndrome (WHS; OMIM #194190) (Hammond et al., 2012) and Smith–Magenis syndrome (SMS; OMIM #182290) (Tomona, Smith, Guadagnini, & Hart, 2006), establishing accurate discriminating characteristics or assisting the determination of phenotype–genotype correlations. In addition, computer‐based models of 3D facial morphology can assist in dysmorphology training (Hammond, 2007).

In the present study, we investigated the effect of CS‐associated mutations in *ABCC9* on craniofacial structures using DSM based analysis of 3D photographs of 20 clinically affected and molecularly proven CS patients. Additionally, we compared facial dysmorphism in CS with genetic disorders that can be a differential diagnosis of CS because of overlapping facial features. Our study assembles the second largest cohort of CS patients studied so far, considering the overall rareness of the disorder (Grange et al., 2019).

We demonstrate that DSM‐based analysis provides a very accurate instrument to classify faces or facial regions along the CS‐control spectrum. In addition, we highlight specific facial differences between CS and genetic disorders with similar dysmorphisms to improve initial diagnosis of these patients. As a result, our findings demonstrate the utility of 3D imaging during a genetic diagnostic process.

## 2 SUBJECTS AND METHODS

### 2.1 Study participants

All data reported here were collected after informed written consent of the patient or the parents or legal guardians for patients younger than 18 years old.

The CS patients group comprised 10 male patients 2.7–16.2 years old and 10 female patients 3.1–22.0 years old. Figure S1 shows distribution of country of origin over age in CS patients. All patients had been molecularly diagnosed with CS either by Sanger‐sequencing of the *ABCC9* gene or whole exome sequencing. All study participants are part of the ICSR and have been reported previously at least once (Grange et al., 2019). 3D face images of individuals with CS were collected during special annual Cantú syndrome research clinics at the Utrecht University Medical Center, the Netherlands, and at Washington University, St. Louis, MO. The accuracy of such 3D imaging devices has been shown to be highly reliable (Aldridge, Boyadjiev, Capone, DeLeon, & Richtsmeier, 2005; Camison et al., 2018). Some images were unusable because of incomplete face coverage or poor subject cooperation. Details of the specific *ABCC9* variants and body weight and height were obtained from the ICSR (Grange et al., 2019).

Images of healthy subjects as well as patients diagnosed with Williams syndrome (WS), Fabry disease (FD), Rubinstein–Taybi syndrome (RTS) and Rasopathies were selected from an existing collection. Dense surface models (DSM) were constructed with the following number of individuals to get the fullest face shape variation possible: Healthy controls included 183 unrelated male patients 0.2–66.5 years old and 173 unrelated female patients 0.3–62.2 years old; WS cohort included 51 unrelated male patients 1–17 years old and 41 unrelated female patients 1.1–19.7 years old; FD cohort included 30 unrelated male patients 3.4–64.4 years old and 38 unrelated female patients 5.8–71.5 years old; RTS cohort included 18 unrelated male patients 2–31 years old and 23 unrelated female patients 1.2–31 years old; Rasopathies cohort included 31 unrelated male patients 1.7–26.4 years old and 40 unrelated female patients 0.9–47.6 years old. However, only age‐ and sex‐compatible subgroups were used during analysis.

For comparison with all male and female CS patients the sample size and age range was as follows: healthy male controls, *n* = 76 (age range: 3.1–21.0 years); healthy female controls, *n* = 69 (age range: 2.4–22.4 years old); male WS patients, *n* = 45 (age range: 2.1–17 years); female WS patients, *n* = 38 (age range: 2.3–19.7 years); male FD patients, *n* = 10 (age range: 3.4–22.7 years); female FD patients, *n* = 18 (age range: 5.8–22.8 years); male RTS patients, *n* = 15 (age range: 2.0–17.9 years); female RTS patients, *n* = 19 (age range: 2.4–21.6 years); male Rasopathies patients, *n* = 15 (age range: 2.4–22.4 years); female Rasopathies patients, *n* = 18 (age range: 2.3–21.9 years old). For comparison with age‐specific male and female CS subgroups the sample size and age range was as follows: male CS subgroups, *n* = 4 (age range: 2.7–5.1 years); *n* = 6 (age range: 10.8–20.8 years); female CS subgroups, *n* = 3 (age range: 3.1–5.5 years); *n* = 7 (age range: 10.0–22.0 years); male healthy controls, *n* = 17 (age range: 3.1–5.8 years); *n* = 36 (age range: 10.0–21.0 years); female healthy controls, *n* = 20 (age range: 2.5–5.8 years); *n* = 25 (age range: 10.0–22.4 years); WS, *n* = 26 (age range: 2.1–5.9 years), *n* = 35 (age range: 10.5–19.7 years); FD, *n* = 3 (age range: 3.4–5.8 years), *n* = 19 (age range: 10.1–22.8 years); RTS, *n* = 11 (age range: 2.0–5.5 years), *n* = 12 (age range: 10.2–21.6 years); Rasopathies, *n* = 18 (age range: 2.3–5.8 years), *n* = 28 (age range: 10.5–22.4 years).

All individuals used in this study are Caucasian but from different countries of origin. Figure S2 shows distribution of country of origin of controls and patients with another disorder used in this study.

Notably, the mutation profile of all age‐matched rasopathies patients is as follows: *HRAS* (*n* = 12), *BRAF* (*n* = 11), *PTNP11* (*n* = 6), *MEK1* (*n* = 2), *KRAS* (*n* = 1), *SOS1* (*n* = 1).

#### 2.1.1 3D image capture and preparation

A total of 20 3D face images of individuals with CS were captured with commercial photogrammetric devices (3dMDface System [3dMD Inc.] and Canfield Vectra 3D system [Canfield Scientific Inc.]). All images were manually annotated by a single operator (PH) with 22 facial landmarks previously shown to be accurate and reproducible (Toma, Zhurov, Playle, Ong, & Richmond, 2009).

### 2.2 DSM building and closest mean classification

A dense surface model of a set of landmarked face surfaces, described in detail elsewhere (Hammond, 2007; Hutton, Buxton, Hammond, & Potts, 2003), comprises shape variation modes arising from a principal component analysis (PCA) of differences of the surface points' positions from those of a comparator average face. Prior to the PCA, using a base mesh (whole face or patch) and aligned sparse landmarks, a dense correspondence of surface points across all faces is induced with no manual interaction. The proportion of face shape variance covered by each DSM mode is computed, and typically the modes are ordered in terms of decreasing variance coverage. For a DSM of a collection of faces of children and adults, the first mode typically reflects facial growth and correlates strongly with age (Hammond, 2007). We retained sufficient modes to cover 99% of shape variance in each constructed DSM.

Each face surface captured has as many as 30,000 mesh points. The *signature* of a face surface is the set of position differences at constituent image mesh points from corresponding points on the mean of age/sex‐matched healthy controls, normalized against the variation in controls. The *signature weight* of a surface is the square root of the sum of the squared normalized differences across all of the densely corresponded points. Signature weight is a rough estimate of facial dysmorphism. A *signature heat map* visualizes the significance of localized differences using a red‐green‐blue spectrum with, for example, red and blue reflecting extreme opposite displacements and green coincidence with the mean of the matched controls. Thus, an axis normal to the face surface reflects inward/outward displacement.

In order to investigate differences in face shape, we used multi‐folded discrimination testing (closest mean; support vector machines; linear discriminant analysis) to determine baseline discrimination rates between controls and syndromes. Anthropometric comparisons were made against appropriate age‐range matched controls.

#### 2.2.1 Comparison of linear regressions

Linear regressions were undertaken for various facial measurements and DSM‐based markers against age. Prism (Graphpad) was used to determine significant differences in slope and/or intercept in comparisons of separate regressions for control, CS and other disorder cohorts.

#### 2.2.2 Statistical analysis

Significance values when comparing gender‐specific facial features of CS patients and controls were calculated using an unpaired Student's *t*‐test throughout the article. Statistical robustness was further enhanced through applying a Bonferroni correction that divides the *p*‐value cut‐off by the number of tests run. To find statistically significant differences in facial features between male and female CS patients and age‐ and gender‐matched controls, 5% *p*‐value cut‐off was divided by 4, resulting in a threshold *p*‐value of .0125.

## 3 RESULTS

We present 3D imaging analysis in 20 CS patients. All patients have confirmed genetic variants in *ABCC9* (Figure S2); in 17/20 patients this variant is de novo. Information regarding genotype and general features of all 20 patients is provided in Table 1. Clinical phenotype among patients was similar to those reported in previous studies. Table S1 lists major clinical features of CS found in the study participants. All patients are part of the ICSR (Grange et al., 2019) and thus have been published at least once before. Patient study numbers applied in this study can be traced back to the ICSR published by Grange et al. (2019), which documents clinical features of each patient in detail.

**Table 1:** Patient *ABCC9* variants and general features (*n* = 20)

Patient	Gender	Age	cDNA variant	Protein alteration	De novo status	Race/ethnic background	Height (cm)	Weight (kg)	Head circumference (cm)	Previous publication
CS0001	M	16	c.3460C>T	p.Arg1154Trp	de novo	C	152	45	56	Grange et al. (2019); Leon‐Guerrero et al. (2016)
CS0002	F	21	c.3461G>A	p.Arg1154Gln	de novo	C	157	44	58	Grange et al. (2019); Leon‐Guerrero et al. (2016); Grange et al. (2006)
CS0005	F	22	c.3460C>T	p.Arg1154Trp	Inherited from affected mother	C	157	68	56	Grange et al. (2019); Leon‐Guerrero et al. (2016)
CS0011	M	11	c.3347G>A	p.Arg1116His	de novo	C	150	47	58	Grange et al. (2019); Leon‐Guerrero et al. (2016)
CS0013	M	2	c.4468T>A	p.Val1490Glu	de novo	C/H	107	18	53	Grange et al. (2019)
CS0016	F	3	c.2378A>T	p.Asp793Val	de novo	C	74	10	51	Grange et al. (2019)
CS0017	F	3	c.3014A>T	p.His1005Leu	de novo	C/H	71	11	48	Grange et al. (2019)
CS0020	M	20	c.3461G>A	p.Arg1154Gln	de novo	C	193	69	56	Grange et al. (2019)
CS0024	M	5	c.4040G>T	p.Arg1347Leu	de novo	C	114	20	53	Grange et al. (2019)
CS0028	F	5	c.3605C>T	p.Thr1202Met	de novo	C/H	114	20	53	Grange et al. (2019)
CS2001	F	11	c.1295 C>T	p.Pro432Leu	de novo	C	138	33	na	Grange et al. (2019); Harakalova et al. (2012)
CS2003	M	3	c.1433C>T	p.Ala478Val	Inherited from affected father	C	69	15	53	Grange et al. (2019); Roessler et al. (2018)
CS2004	M	12	c.178C>T	p.His60Tyr	de novo	C	137	36	na	Grange et al. (2019)
CS2005	M	17	c.3460C>T	p.Arg1154Trp	de novo	C	na	50	na	Grange et al. (2019)
CS2008	F	15	c.3346C>G	p.Arg1154Gly	de novo	C	na	na	na	Grange et al. (2019)
CS2009	F	20	c.3461G>A	p.Arg1154Gln	de novo	C	na	na	na	Grange et al. (2019)
CS2010	F	17	c.3460C>T	p.Arg1154Trp	de novo	C	183	57	57	Grange et al. (2019)
CS2012	M	10	c.3347G>A	p.Arg1116His	Inherited from affected mother	C	na	na	na	Grange et al. (2019); Harakalova et al. (2012); Roessler et al. (2018)
CS2013	F	10	c.3460C>T	p.Arg1154Trp	de novo	C	127	26	na	Grange et al. (2019); Harakalova et al. (2012)
CS2014	M	3	c.3461G>A	p.Arg1154Gln	de novo	C	91	15	51	Grange et al. (2019)

Abbreviations: C, Caucasian; H, Hispanic; na, not assessed.

### 3.1 Patient demographics

The 20 patients (10 females and 10 males) range in age from 2.7 to 22.0 years (mean: 11.46 years) (Table 1). Patients were recruited by two clinical genetic centers, in the Netherlands and the United States. All participants are Caucasian and most (17/20) identify as non‐Spanish/Hispanic/Latino, reflecting the location of the participating sites.

### 3.2 Facial dysmorphology in CS

Using software developed in house, a dense surface model (DSM) was constructed from the landmarked images including 20 CS patients, 272 individuals with a syndrome other than CS (203 age‐matched) and 356 controls (145 age‐matched). Approximately 25,000 points on each face were used in the dense correspondence. The corresponding DSM contained 40 principal components covering 99% of all shape variation.

**Figure 1:**
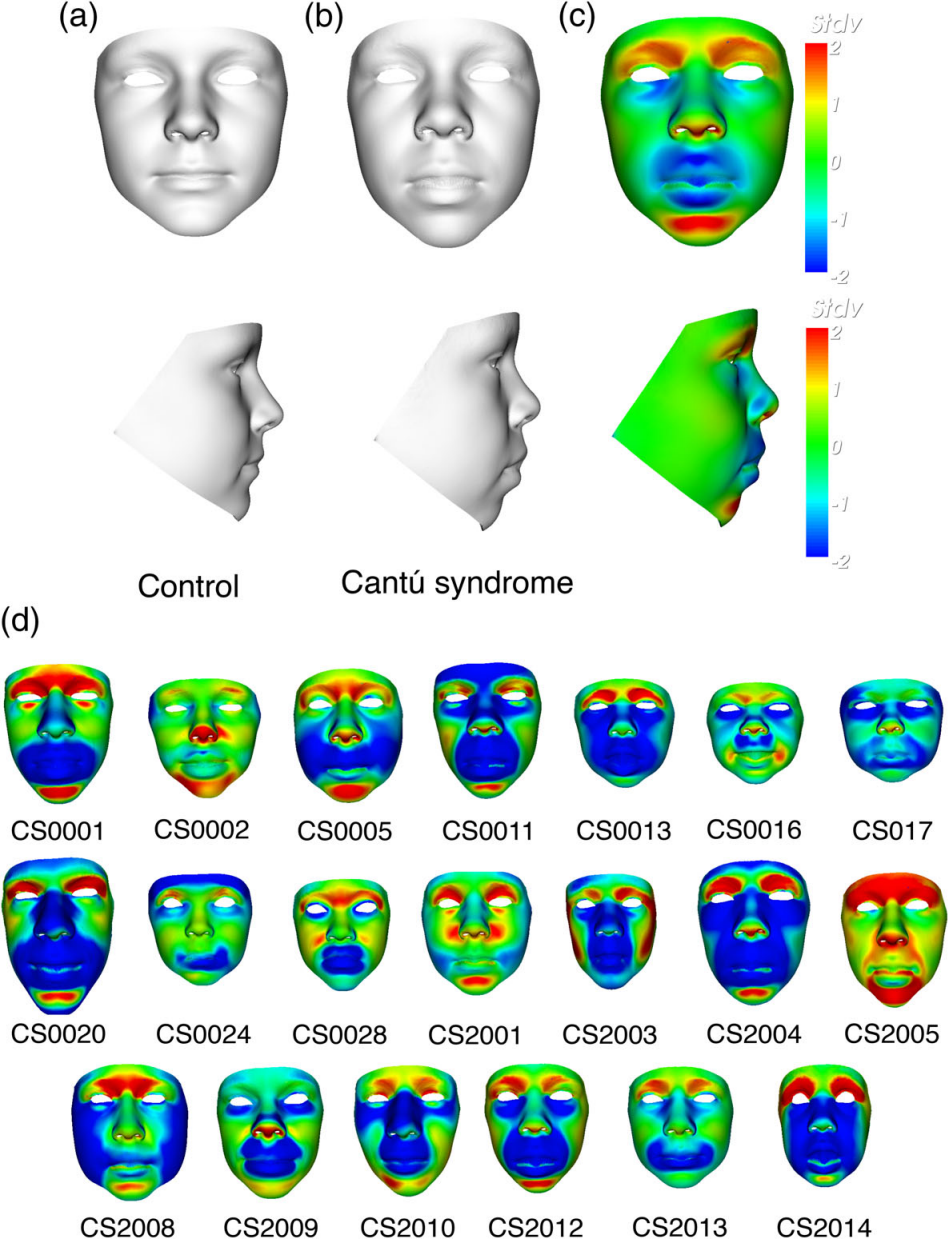
Three‐dimensional images of 20 Cantú syndrome patients. Portrait and profile of average faces of 20 individuals with Cantú syndrome (b) and 143 healthy controls (a) with same mean age. (c) Heat‐mapped face signatures showing normalized face shape differences (displacement of surface points orthogonal to face surface and parallel to three axes) compared with matched control mean. The red/green/blue coloring reflects displacement orthogonal to the surface in the first signature (red/green/blue = contraction/coincidence/expansion) and in the three axial signatures parallel to the colored arrows with green = no displacement and maximal red‐blue hues reflecting two SDs or more. (d) Heat mapped face asymmetry signatures of all investigated 20 Cantú patients [Color figure can be viewed at http://wileyonlinelibrary.com]

Comparison of the mean faces of the CS and control group confirms many of the previously published facial features. In Figure 1, the mean face of the 20 CS patients (b) and an age‐matched control (a) are shown in portrait and profile. A signature heat map of the mean CS face reflects location differences between corresponding points on the mean age‐matched control along a surface normal (Figure 1c). Points on the CS mean face are shown in green if they are coincident with their corresponding points on the unaffected mean surface. They are colored red/blue if they are displaced inward/outward relative to the matched mean control surface on a − 2 to +2 *SD* scale. The abundance of blue especially on the nose, lips and mandible of the mean CS face shows clear differences compared to controls. Signature heat maps of all 20 CS patients can be found in Figure 1d.

When compared with the age‐matched control mean, the mean CS face shows general lengthening of the face, whereas width seems to be normal. The nasal bridge appears broader and the nasal tip turns upwards resulting in anteriorly facing nostrils. The philtrum length is increased and the lips show general thickening as well as concave curling of the upper lip. Additionally, epicanthic folds are present resulting from narrowing of both the supra‐orbital arch and notch. Periorbital fullness is vaguely present. Notably, due to presence of eyelashes and wetness of eye surfaces, 3D camera devices often lead to image capture errors, such as holes in the surface or even spikes, or do not obtain an accurate surface around the eyes, at all.

All DSM analyses were performed for a reduced face patch since ears were not included during 3D image capture. However, from previous studies we know that no dysmorphologies of the ears are expected (Grange et al., 2019).

### 3.3 Mean male and female faces and dynamic morphs

**Figure 2:**
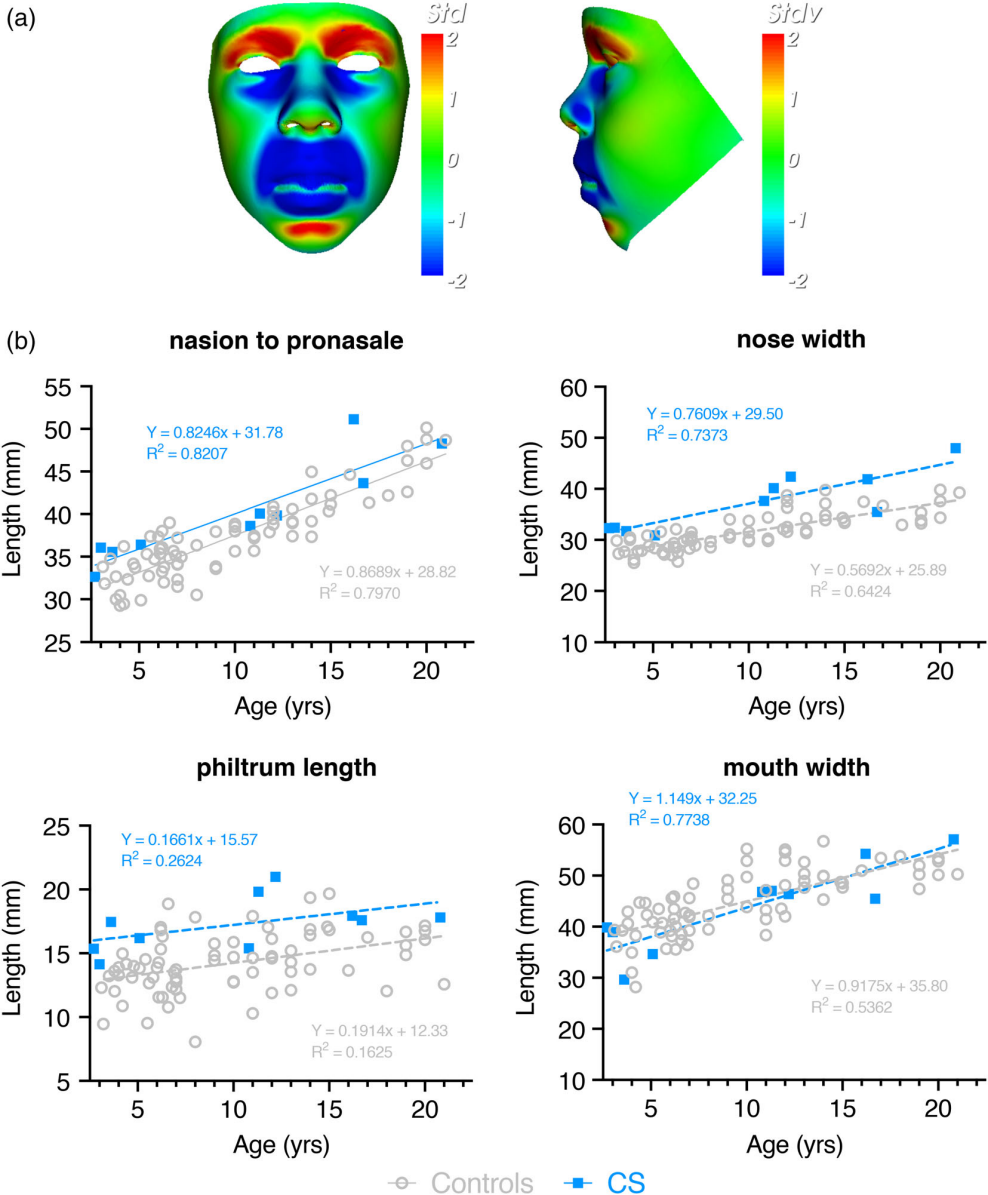
Male‐specific facial abnormalities in CS patients. (a) Portrait and profile views of the color‐coded comparison of male CS patients with the average of the male control group (*n* = 76, age range: 3.1–21 years). The color scale of all depicted heatmaps is equal to Figure 1. (b) A scatter of age against different facial features is shown for a single DSM for a reduced face patch (no ears) for both age‐matched controls and male CS patients. Statistically significant differences between male CS patients and age‐ and gender‐matched controls confirm a wider nose (*p* = .0003), longer philtrum (*p* = .0004) and increased nose length (*p* = .011). DSM, dense surface model [Color figure can be viewed at http://wileyonlinelibrary.com]

**Figure 3:**
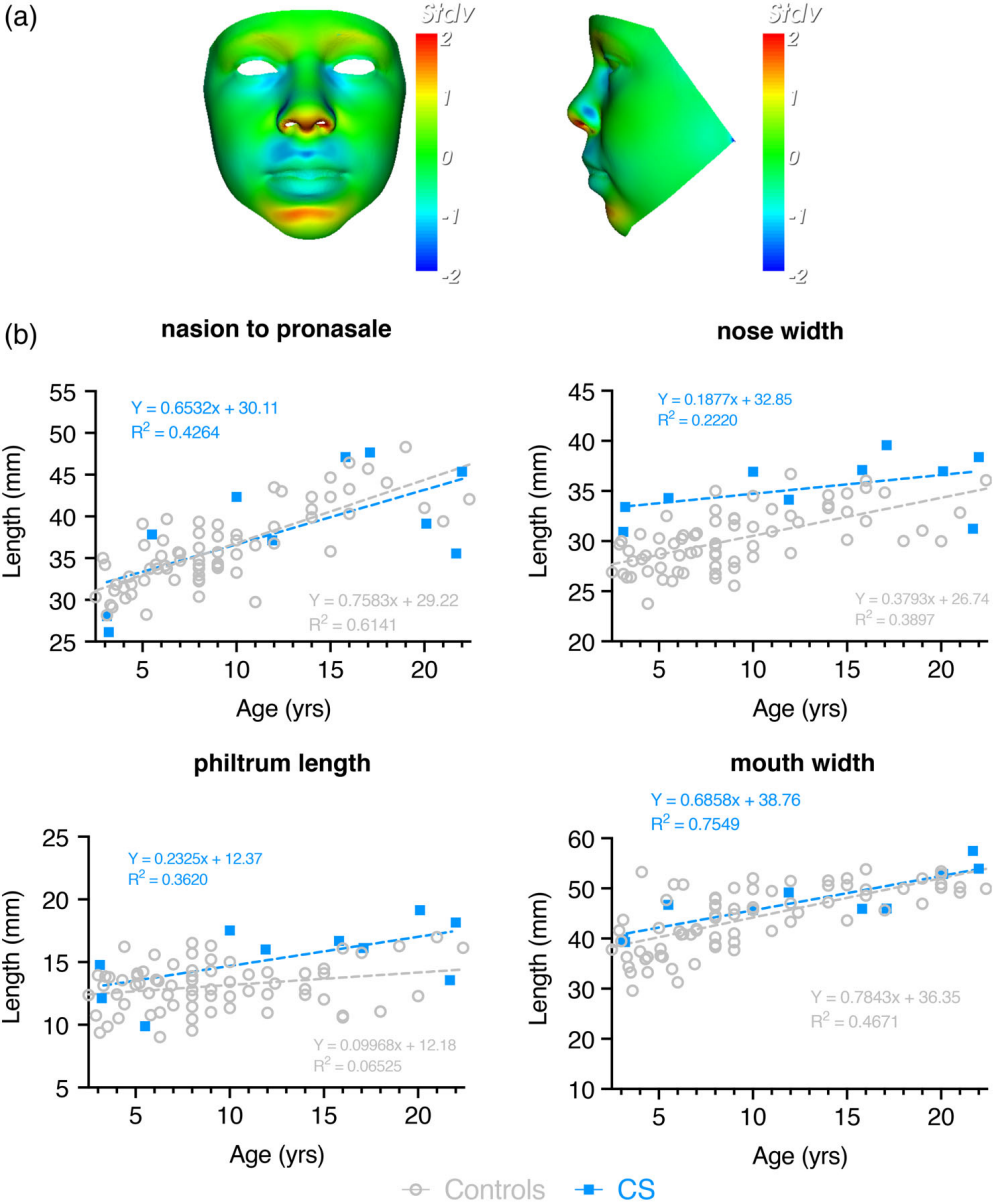
Female‐specific facial abnormalities in CS patients. (a) Portrait and profile views of the color‐coded comparison of female CS patients with the average of the female control group (*n* = 69, age range: 2.4–22.4 years). The color scale of all depicted heatmaps is equal to Figure 1. (b) A scatter of age against different facial features is shown for a single DSM for a reduced face patch (no ears) for both age‐matched controls and female CS patients. Statistically significant differences between female CS patients and age‐ and gender‐matched controls confirm a wider nose (*p* = .0003). DSM: Dense surface model [Color figure can be viewed at http://wileyonlinelibrary.com]

Figures 2 and 3 show static portrait and profile views signature heat map comparisons between all CS males (*n* = 10) (Figure 2) or females (*n* = 10) (Figure 3) and an age‐ and sex‐matched average of appropriate controls. Dynamic morphs of portrait and lateral views between the control and CS mean faces very clearly demonstrate the important differences in facial characteristics in both males and females (see Supplementary Movie S1 and S2).

When compared to the mean face of the male controls, the color‐distance‐coded comparison of the mean CS male suggests a longer and wider nose, a longer philtrum, retrognathia, fuller lips, a more bulbous nose, a more prominent and pointed chin and lifting of supra‐orbital ridges (Figure 2a). By viewing the dynamic comparisons of the mean faces these face‐shape differences are shown in a more dramatic manner. Additionally, a lengthening of the entire face becomes visible. Figure 2b shows the results of a double‐tailed *t*‐test comparison where this is appropriate. Statistically significant differences between male CS patients and age‐ and gender‐matched controls confirm the wider nose (*p* = .0003), longer philtrum (*p* = .0004) and increased nose length (*p* = .011), whereas there is no obvious increase in mouth width.

The static comparison (Figure 3a) as well as the dynamic morphs in the Supplementary Material show similar but less pronounced facial differences in the female cases. Whereas, patients show an upward/backward displacement of the nose resulting in anteriorly facing nostrils compared to the control group, both the lengthening of the face and therefore nose as well as thickening of the lips are less significant. Additionally, the chin seems less pointed. Both male and female individuals show no obvious increase in mouth width. The nose width (*p* = .0003) is statistically significant when compared to age‐ and gender‐matched controls, whereas philtrum length (*p* = .032), nose length (*p* = .325) and mouth width (*p* = .092) are not (Figure 3b).

In order to compensate for the wide age range (2–22 years) and the changes in morphometric analysis according to age, we additionally investigated differences in facial features in the following age‐ and gender‐specific CS subgroups compared to respectively matched controls: Male subgroup 1 (age 2–5 years), male subgroup 2 (age 10–22 years), female subgroup 3 (age 2–5 years) and female subgroup 4 (age 10–22 years). Signature heat map comparisons between all four subgroups and controls suggest that the pointiness of the chin develops at a later age (>10 years) whereas periorbital fullness decreases over time (Figure S4a‐S7a). Retrognathia, lifting of supra‐orbital ridges and bulbousness of the nose develop early on. Whereas younger male CS patients show a significantly wider nose (*p* = .0026), longer philtrum (*p* = .0087) and increased nose length (*p* = .0079) compared to healthy individuals, older male CS patients only reveal a statistically significant difference in nose width (*p* = .0061) and philtrum length (*p* = .0112) when compared with age‐ and gender‐matched controls suggesting a slight decrease or at least stagnation in overall dysmorphism over age (Figure S4b and S5b). Similary, nose width of female CS patients in young age shows statistical significance (*p* = .0101), but becomes more equal to healthy individuals in older age (*p* = .079). Philtrum length, on the other hand, is only significantly increased in older female CS patients (*p* = .0036) (Figure S6b and S7b). Notably, sample sizes in these subgroups are restricted and results should therefore only be seen as indications of progression of facial anomalies in CS.

### 3.4 Facial growth and overall dysmorphism in CS

**Figure 4:**
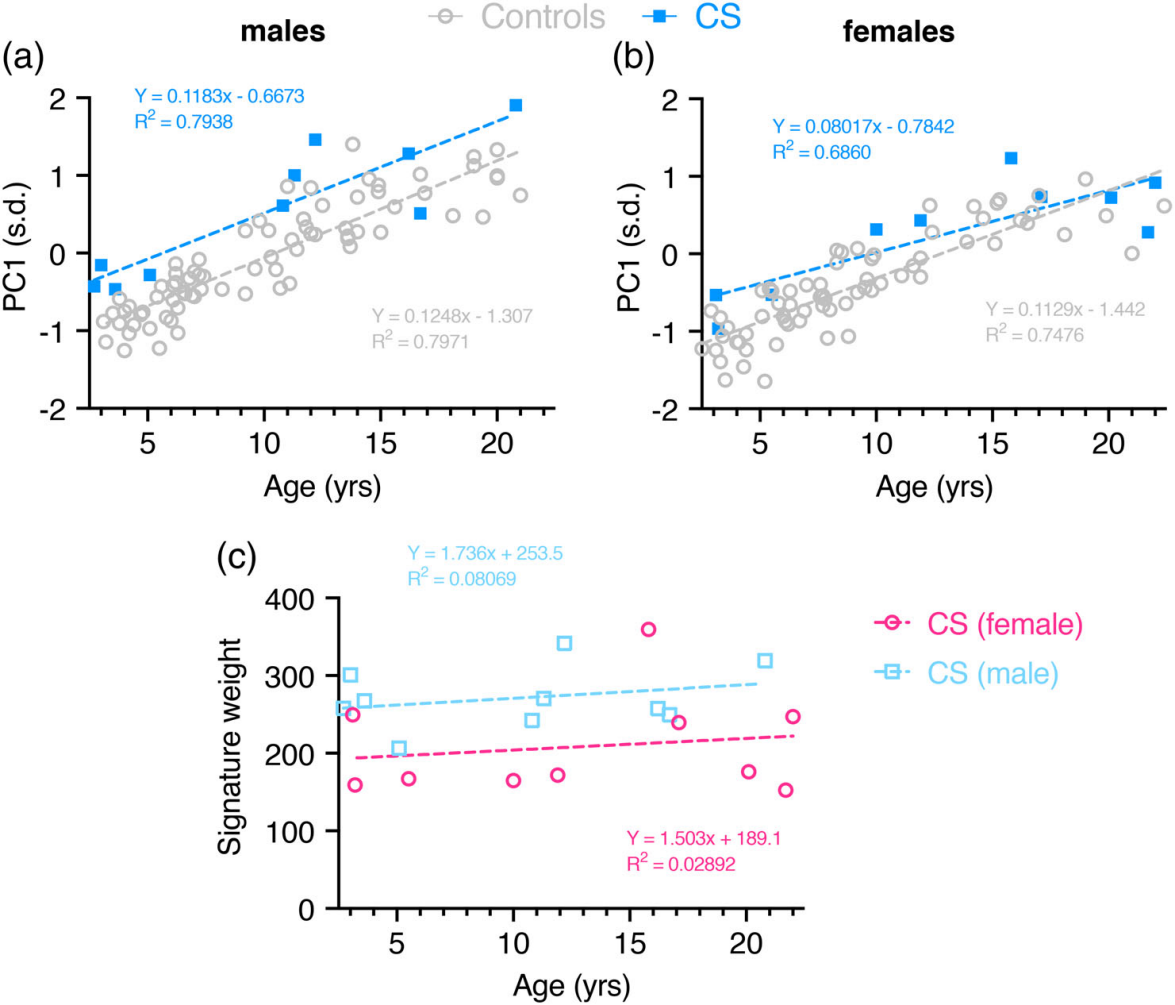
Facial growth and overall dysmorphism in CS. (a) A scatter of age against PC1 is shown for a single DSM for a reduced face patch (no ears) for both the control and CS subgroups. The scatter is annotated by separate linear regression lines for each subgroup. PC1 reflects facial growth. Male control group includes *n* = 76 individuals with an age range of 3.1–21.0 years; female control group includes *n* = 69 subjects ranging from 2.5 to 22.4 years of age. (b) A scatter of age against signature weight is shown for a single DSM for a reduced face patch (no ears) for male and female CS subgroups. Signature weight is a rough estimate of facial dysmorphism. DSM, dense surface model; PCA, principal component [Color figure can be viewed at http://wileyonlinelibrary.com]

In a face DSM with mixed age range, the first principal component (PC1) typically reflects growth and is highly correlated with age. Separate linear regression of PC1 against age for gender‐specific control and CS subgroup (2–22 years) suggests face size is significantly greater in patients (males: *p* < .0001; female: *p* = .0325) (Figure 4a). However, care must be taken in this interpretation as the retrognathia and open bite may exaggerate facial length in CS patients. On the other hand, a majority of CS patients show increased body size and macrocephaly at birth, hence an increased facial growth compared to controls is to be expected. Additionally, this reflects observations stated when comparing the average face of CS patients and controls (Figure 1a,b).

When calculating PC1 for all four age‐ and gender‐specific subgroups facial growth is still significantly increased in both male subgroups (age range 2–5 years: *p* = .0004, age range 10–22 years: *p* = .001), whereas facial growth seems to become more similar to healthy controls in female CS patients (age range 2–5 years: *p* = .0713, age range 10–22 years: *p* = .0776) (Figure S8a,b).

The overall dysmorphism score was calculated as the square root of the sum of squared differences of displacements in the signature heat map comparison with the mean of age‐ and sex‐matched controls. Firstly, this score suggests that facial dysmorphism does not increase much over time in CS patients (Figure 4b). However, a comparison between female and male patients shows significantly more dysmorphism across all ages in male patients (*p* = .0151). Calculation of signature weight in all four age‐ and gender‐specific subgroups confirms these observations (Figure S8c).

### 3.5 DSM analysis results in high levels of discrimination between CS and other disorders with similar facial dysmorphologies

According to previous publications (Grange et al., 2019), many CS patients initially received other diagnoses, often based on coarse facial features. This has been extremely stressful for parents who were informed about a bad prognosis based on a wrong diagnosis. To evaluate whether 3D imaging analysis can be successfully applied to discriminate between disorders and thus avoid misdiagnoses, we compared facial features of CS patients with readily available cohorts of other genetic disorders with facial anomalies that have been mistaken with CS‐related anomalies in the past; namely Fabry disease (FD) (*n* = 28) (Cox‐Brinkman et al., 2007), Rasopathies (*n* = 33) (Hammond et al., 2004), Williams syndrome (WS) (*n* = 83) (Hammond & Suttie, 2012) and Rubinstein–Taybi syndrome (RTS) (*n* = 34) (Menke et al., 2018). Notably, mucopolysacharidosis is the most common misdiagnosis in CS based on similar facial appearance (Grange et al., 2019), however there was no collection of 3D images from a patient cohort available for comparison. For this purpose, subgroups of individuals with other syndromes were selected in terms of age‐range compatibility with the CS patients. Any individual's face can be compared to variation between the average face of two subgroups.

**Figure 5:**
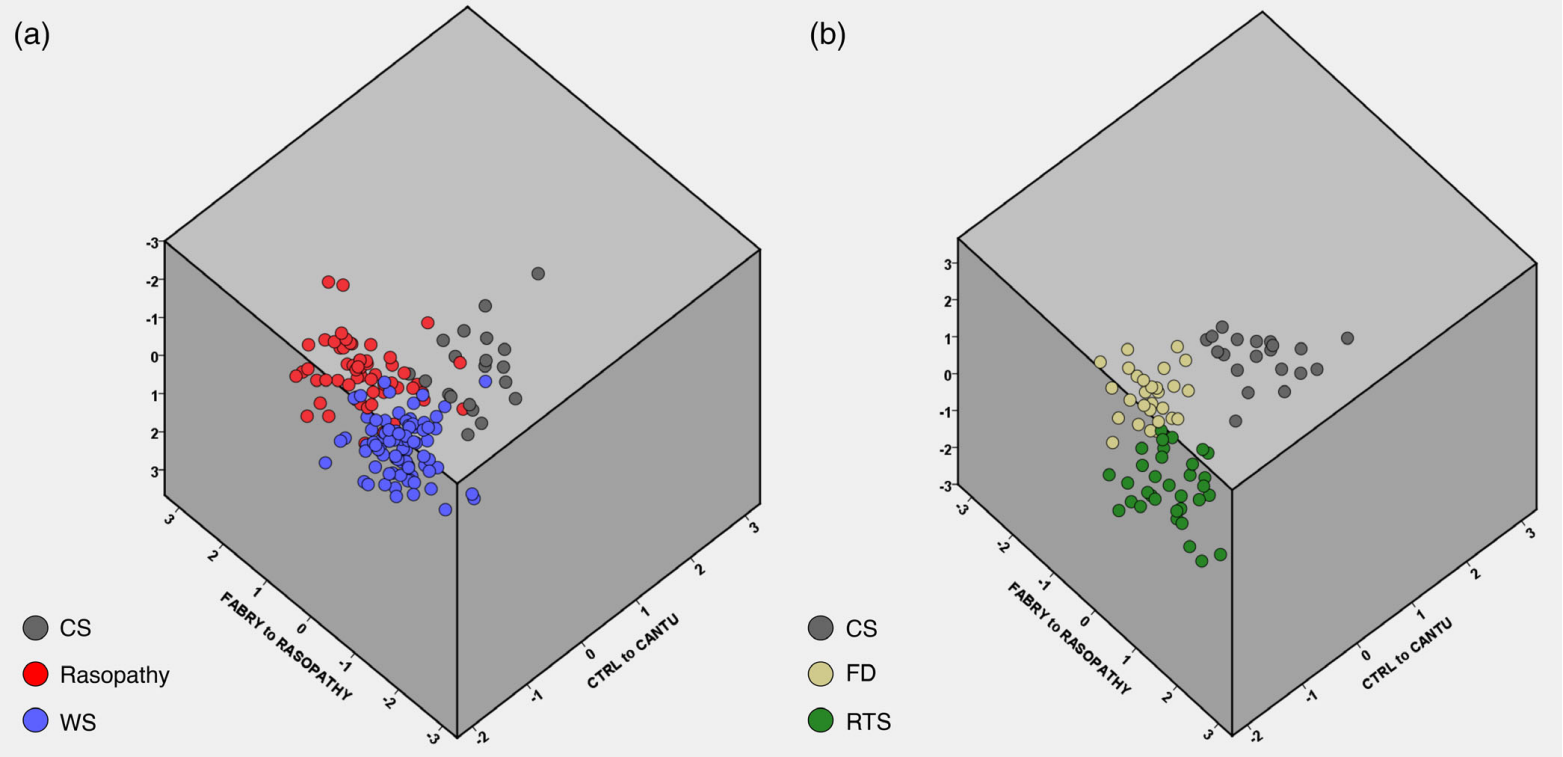
Comparison Of Mean Faces Along 3 Axes. Comparison between mean control to mean CS; mean FD to mean Rasopathy and mean RTS to mean WS. (a) CS, Rasopathy and WS are depicted. (b) CS, FD and RTS are depicted. Sample size, CS, *n* = 20 (age range: 2.7–22.0 years); Rasopathies, *n* = 33 (age range: 2.3–21.9 years); WS, *n* = 83 (age range: 2.1–19.7 years); RTS, *n* = 59 (age range: 2.0–21.6 years); FD, *n* = 28 (age range: 3.4–22.8 years). CS, Cantú syndrome; FD, Fabry disease; RTS, Rubinstein–Taybi syndrome; WS, Williams syndrome [Color figure can be viewed at http://wileyonlinelibrary.com]

Figure 5 shows a simultaneous comparison along three axes: mean control to mean CS; mean FD to mean Rasopathy and mean RTS to mean WS. No overlap could be observed between the CS, FD and RTS subgroups, but CS does share small similarities with Rasopathy and WS patients.

Separate linear regressions of PC1 against age for CS and the remaining disorder subgroups suggest both delay and diminished rate of facial growth in all syndromes but CS. The difference in intercept was highly statistically significant (CS/Rasopathies and CS/RTS, *p* < .0001; CS/FD, *p* = .0021). When comparing CS to WS the slopes differ significantly (*p* = .0014), but it was not possible to test whether the intercepts differ significantly (Figure S9). These results are to be expected considering the universal clinical observation of generalized macrosomia, with large birth weight and length, as well as persistent macrocephaly commonly reported in CS. In contrast, individuals with WS (Carrasco, Castillo, Aravena, Rothhammer, & Aboitiz, 2005), FD (Colon et al., 2017) and Rasopathies (Tajan, Paccoud, Branka, Edouard, & Yart, 2018) generally show low birth weight. Similar observations could be made when comparing age‐specific CS subgroups (subgroup 1: age range 2–5, subgroup 2: age range 10–22) to age‐matched disease cohorts (Figure S10).

Calculation of the overall dysmorphism score for each individual and separate linear regressions against age additionally suggest that facial dysmorphism increases with age in all disorders but CS. The difference in slope was highly statistically significant when comparing CS and Rasopathies (*p* = .0057). No statistical significance was found in all other cases (CS/WS, *p* = .3; CS/RTS, *p* = .0862; CS/FD, *p* = .2780) (Figure S11). When separating CS patients in subgroups according to age, overall dysmorphism of young CS patients is significantly decreased compared to individuals with WS (Figure S12).

## 4 DISCUSSION

In this study, facial dysmorphism in patients with CS is objectively assessed by 3D dense surface modeling and anthropometric analysis, revealing facial dysmorphic features in male and female patients. The most prominent facial anomalies shared by both genders are a broadened nasal bridge and wider nose, narrow supra‐orbital arches, epicanthal folds, a longer philtrum, thick lips, and retrognathia. Male patients additionally reveal lengthening of the face and nose which is less pronounced in females who, on the other hand, show an upward and backward displacement of the nose, anteverted nares, and lifting of supra‐orbital ridges. The majority of all CS‐related facial features seem to develop early on. Merely, the pointed chin and the longer philtrum in female patients are only revealed in older age. Facial anomalies tend to remain until the age of at least 22 with only the nose width of female CS patients normalizing after childhood.

Face size and growth in CS patients, interpreted by PC1 in the model, is always greater compared to age‐matched controls. Overall facial dysmorphism, is significantly higher in male CS patients compared to females but unlike the other syndromes investigated, it barely increases or even stagnates with age in all studied subgroups. Notably, we would like to point out that all analysis performed in the four age‐ and gender‐specific CS subgroups can merely be seen as an indication of facial development in CS since the subject numbers are small (3–7 patients) and therefore miss statistical robustness. Thus, we strongly suggest to primarily focus on data aquired from CS subgroups containing all male or female patients as shown in Figures 2 and 3. In order to properly assess changes in facial features in different CS age groups one would require a cohort with significantly more subjects.

Our results are mostly in line with the features described in various clinical reports that assessed facial characteristics of CS patients based on medical photography (Roessler et al., 2018; Scurr et al., 2011). However, increase in mouth width which has been previously reported in the literature (Grange, Nichols, & Singh, 2014; Roessler et al., 2018) was not observed applying 3D image analysis. Hence, we demonstrate that 3D imaging recognizes common facial anomalies observed in CS patients. Since the results generated in this study are produced by objective quantitative analysis and are thus not hampered by inter‐observer variability, the applied approach enables a more reliable assessment and quantification of facial anomalies observed in CS patients. Moreover, we include comparison of both male and female CS patients with respective controls as well as evaluation of facial growth and overall dysmorphism.

Notably, our study cohort only includes Caucasians, originating from both the Netherlands and the United States with an age range of 2.7–22.0 years, which resembles the overall patient demographic reported in literature so far (Grange et al., 2019). Even though all applied control subjects in this study are also Caucasian, they originate from two European populations of close phylogenetic and geographic proximity, the UK and The Netherlands (Figure S2). Even though previous studies have shown facial differences in different adult Caucasian populations (Hopman, Merks, Suttie, Hennekam, & Hammond, 2014), we do not expect these to be present in younger individuals.

The uneven age distribution of patients, which can be observed in our CS cohort, might be seen as a limitation when making simple anthropometric comparisons with controls but for shape specific considerations like face signature each individual is normalized against a sex‐and‐age matched set of controls which is more reliable.

It would be of interest to perform a similar study in CS patients with an increased age as well as from non‐European ancestry, which would require a larger cohort as is identified currently. Additionally, 3D face morphometry of CS patients could be compared to healthy siblings as similarity and variability between healthy and diseased siblings might highlight the real face morphometry of the disorder. This, however, would require capturing 3D images of healthy siblings of the same sex and a similar age which might be a challenge given the overall rarity of CS.

Another interesting prospect of 3D face shape models is the appliance in genotype–phenotype studies. So far, no obvious differences in frequency or degree of craniofacial dysmorphology, hypertrichosis, or skin appearance depending on the site of the *ABCC9* variant could be observed in CS individuals. Also, cardiovascular and neurological anomalies were similar across the *ABCC9* variant locations (Grange et al., 2019). Even within families containing multiple affected members, there is highly variable expression of CS features, comparable with other autosomal dominant conditions (Grange et al., 2019; Roessler et al., 2018). 3D imaging technologies offer a way to investigate genotype–phenotype correlations in terms of facial features. However, one would have to increase cohort size in order to a perform reliable analysis.

Notably, it would be interesting to acquire and analyze 3D facial photographs of patients harboring a variant in *KCNJ8* because they may potentially have phenotypic differences that can only be distinguished by quantitative evaluation instead of by eye. However, since at the moment only two patients with a CS‐associated variant in *KCNJ8* are known (Brownstein et al., 2013; Cooper et al., 2014) this would be of interest once a larger cohort has been acquired in the future. Notably, it is speculated that such variants might frequently be lethal (Grange et al., 2019).

Clinical geneticists often refer to facial differences and features that offer clues to diagnosis. Articulating those differences can be challenging, especially if an individual's face shows a mild facial phenotype or if morphological characteristics are manifested in only part of the face. These complications can lead to misdiagnosis of patients. According to a recent report on 74 patients from the ICSR, 41% of participating CS patients initially received other diagnoses based on various primarily assessed features, including a lysosomal storage disorder such as mucopolysaccharidosis or Pompe disease based on neonatal cardiomegaly (Grange et al., 2019). The majority of misdiagnoses were based on facial dysmorphology and hypertrichosis and could however not be confirmed by additional genetic of metabolic testing. Since these misdiagnoses implicate a completely different prognosis and life expectancy, earlier CS face recognition could prevent unnecessary stress for parents.

By comparing facial characteristics of CS with patient cohorts whose facial anomalies have been mistaken with CS in the past we demonstrate that 3D imaging analysis can be successfully applied to delineate between syndromes and thus avoid further misdiagnoses. It would be of interest to perform a similar comparison between CS and MPS patients in the future. Notably, congenital hypertrichosis, also observed in all CS patients (Grange et al., 2019), can sometimes be applied as clear discriminator between CS and other mentioned syndromes, if severe enough.

Most importantly, the described 3D imaging technique reveals multiple advantages including practicality, availability and low costs. 3D cameras and appropriate computer software for analysis are available in every clinic that makes assessing facial morphology via 3D imaging quick and cheap. Hence, the implementation of this in general practice for clinical diagnosis of CS patients but also other disorders with a facial component is feasible.

Recent innovations based on 2D technologies combined with computer vision and deep learning algorithms also hold promise to be worthwhile in diagnosis of genetic disorders with facial components (Gurovich et al., 2019; van der Donk et al., 2019). Although 2D images are more readily available, they are subject to greater variation in quality and pose due to projection distortion, and more sophisticated analysis is required to extract the discriminating features. Thus, a significantly increased number of images is required to train the pattern recognition algorithms. Therefore, 2D technologies may represent a considerable alternative in clinics to successfully diagnose CS and other genetic disorders with facial anomalies in the future, but it still remains to be seen which approach has more potential to be established in a stable fashion.

## 5 CONCLUSION

In conclusion, we report a valid method for quantification of facial dysmorphic features in CS.

Although facial dysmorphism in CS can be clinically recognizable, an objective, quantitative evaluation is especially valuable when assessing phenotypically or genotypically unusual cases.

The genes associated with CS have only recently been identified. Hence, we suspect there may be more affected individuals—particularly older patients—who have not received genetic testing and therefore remain undiagnosed. We demonstrate that 3D imaging analysis can be confidently applied to unravel characteristic facial features of CS and successfully discriminate between the syndrome and other genetic disorders it has been confused with in the past due to facial similarities. This will help in diminishing parental anxiety related to misdiagnoses.

In the future, 3D‐based analysis will enable the recognition of CS facial characteristics in patients who have not had an adequate clinical diagnosis. Subsequent molecular analysis of such patients may identify mutations and in combination with detailed facial analysis enable unequivocal identification of causative genes. Considering the current efforts to develop a pharmacological treatment for CS (Ma et al., 2019; McClenaghan et al., 2019), 3D technology additionally may enable the evaluation and quantitative measurement of effective response to therapy options in the future which will possibly lead to a decrease in coarseness of the face.

## CONFLICT OF INTEREST

The authors declare no conflict of interest.

## AUTHOR CONTRIBUTIONS

Research clinic activities were coordinated by HIR, KS, DKG, GvH, and MMvH. HIR, PH, and MMvH designed the study and formulated the research question. 3D imaging analysis was conducted by HIR and PH. HIR wrote the initial manuscript draft, and PH and MMvH reviewed and revised the manuscript. DKG, NVAMK, and GvH critically reviewed the manuscript. GvH provided funding acquisition.

## Supporting information


**Video S1**

**Video S2**

**Appendix** S1: supporting information

## Data Availability

The data that support the findings of this study are available from the corresponding author upon reasonable request.
