# Supplementary material for: Three‐dimensional facial morphology in Cantú syndrome
Source: Am J Med Genet A. 2020 Feb 26;182(5):1041–52. doi: 10.1002/ajmg.a.61517 (PMC7217184; doi:10.1002/ajmg.a.61517)
Supplement: Supplementary file 3 — Appendix S1: supporting information [file AJMG-182-1041-s003.docx]

**Supplementary Information**

**Supplementary Figure 1**

**Distribution of country of origin over age in CS patients.** (a) Male CS patients. (b) Female CS patients.

**
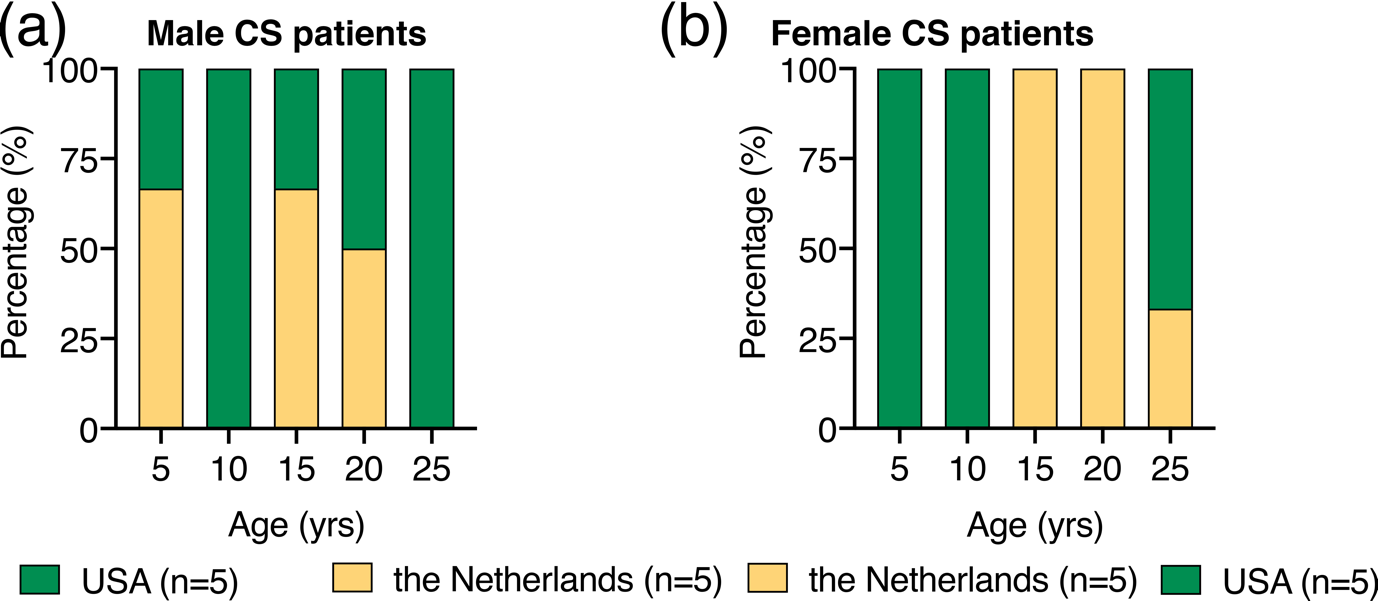
**

**Supplementary Figure 2**

**Distribution of country of origin over age.** (a) Healthy controls. (b) Individuals with genetic disorders that have been mistaken with CS in the past. Individuals depicted within bars fully coloured represent age-matched subjects used for analysis only. Individuals within bars with pattern represent subjects only used for construction of DSM. Individuals within bars fully coloured represent age-matched subjects used for further analysis. Subject numbers refer to all individuals used within the study.


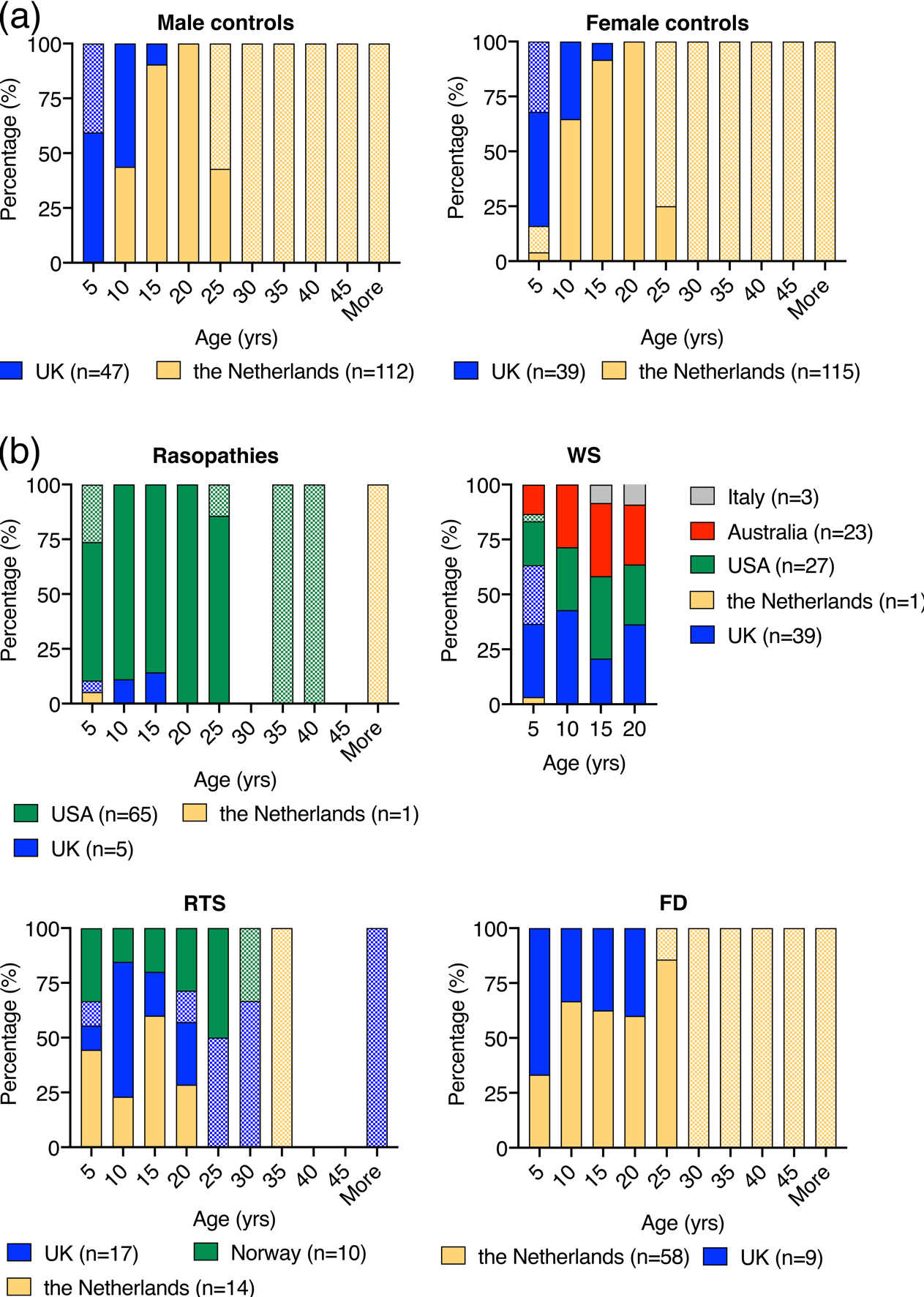


**Supplementary Figure 3**

**Genotype participating individuals with CS (n=20).** Schematic of SUR2 protein structure including domains: TMD0, transmembrane domain 0, with five predicted transmembrane helices; TMD1, with six transmembrane helices; NBD1, nucleotide-binding domain 1; TMD2, with six transmembrane helices; and NBD2. All mutated residues are indicated. Variants occurring in more than a single patient are only shown once. Number of patients harbouring each variant is indicated in brackets. Transmembrane domains (TMDs) are highlighted in light green, nucleotide-binding domains (NBDs) in dark green.


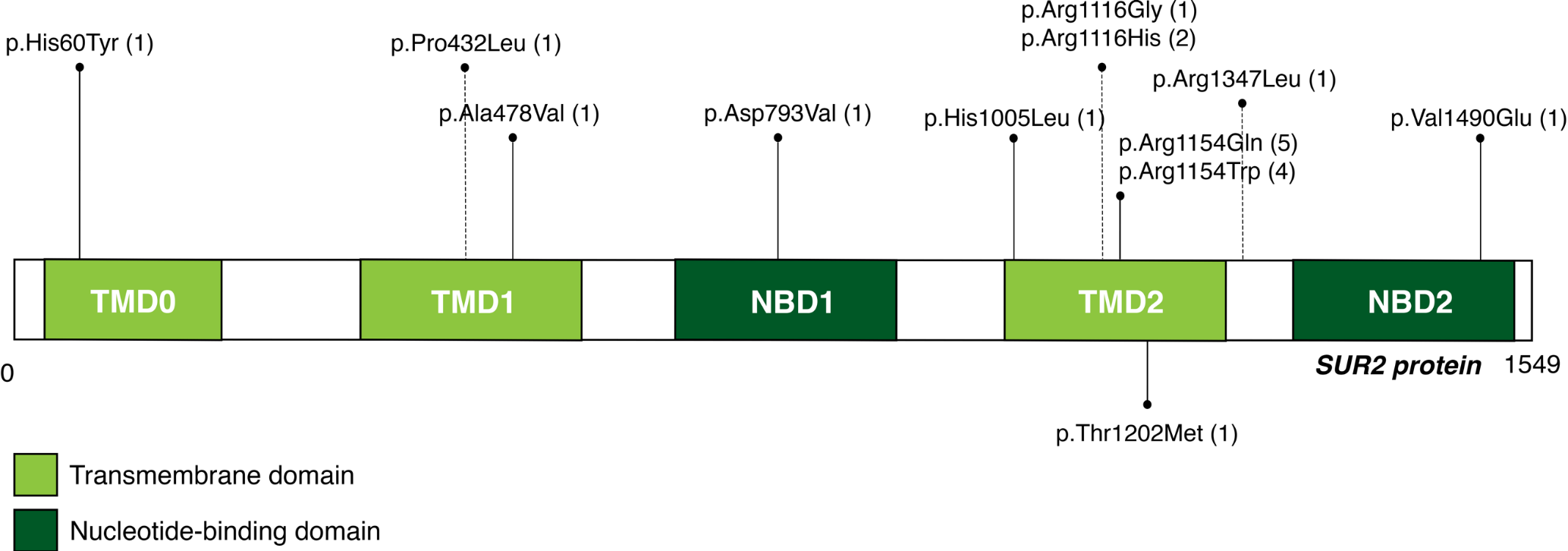


**Supplementary Figure 4**

**Facial abnormalities in male CS patients from 2-5 years of age. (a)** Portrait and profile views of the colour-coded comparison of male CS patients (n=4, age range:2.7-5.1 years) with the average of the male control group (n=17, age range: 3.1-5.8 years). The colour scale of all depicted heatmaps is equal to Figure 1. **(b)** A scatter of age against different facial features is shown for a single DSM for a reduced face patch (no ears) for both age-matched controls and male CS patients. Statistically significant differences between male CS patients and age- and gender-matched controls confirm a wider nose (p=0.0026), longer philtrum (p=0.0087) and increased nose length (p=0.0079).

DSM: Dense surface model.

**
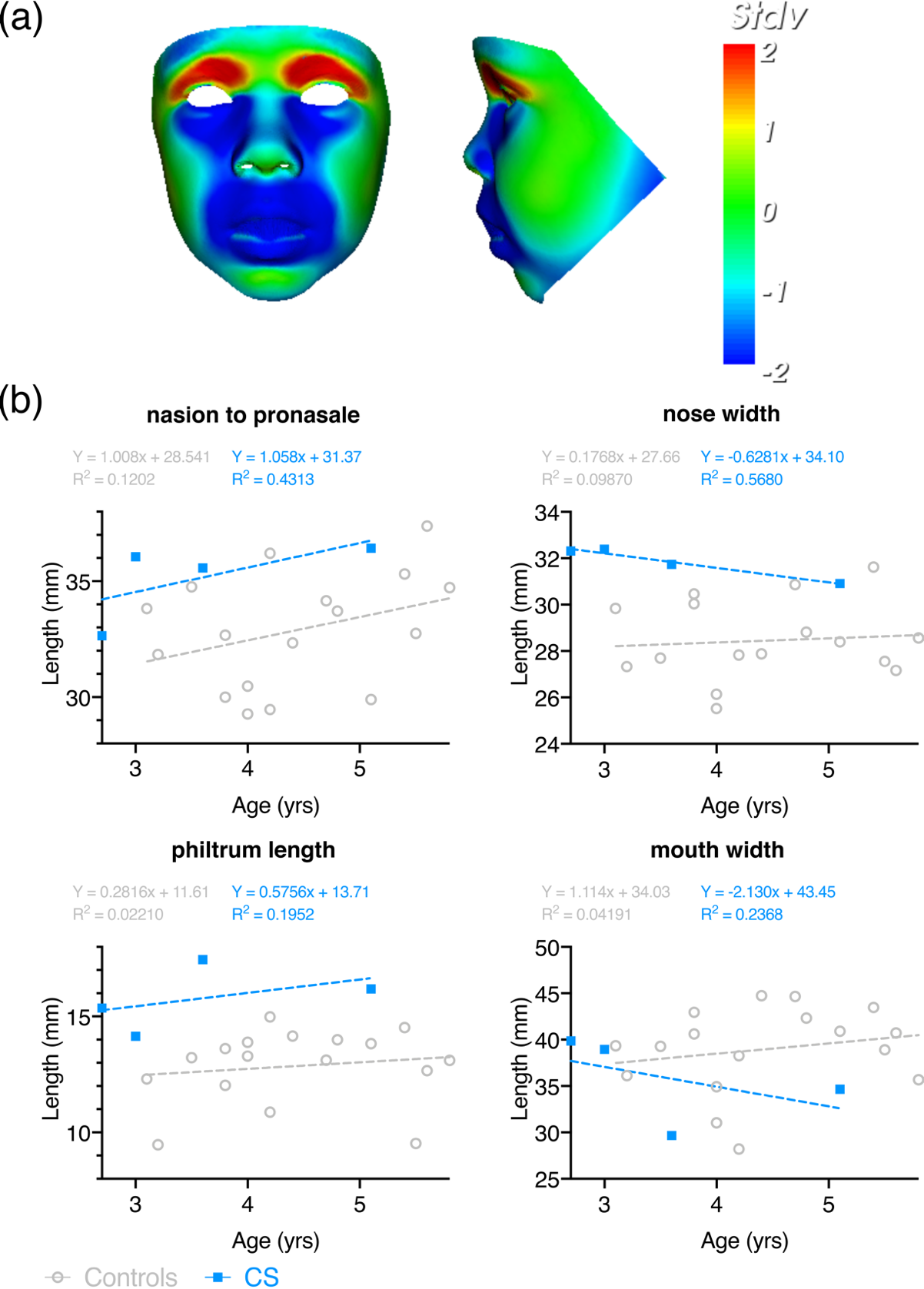
**

**Supplementary Figure 5**

**Facial abnormalities in male CS patients from 10-22 years of age. (a)** Portrait and profile views of the colour-coded comparison of male CS patients (n=6, age range:10.8-20.8 years) with the average of the male control group (n=36, age range: 10.0-21.0 years). The colour scale of all depicted heatmaps is equal to Figure 1. **(b)** A scatter of age against different facial features is shown for a single DSM for a reduced face patch (no ears) for both age-matched controls and male CS patients. Statistically significant differences between male CS patients and age- and gender-matched controls confirm a wider nose (p=0.0061) and longer philtrum (p=0.0112).

DSM: Dense surface model.

**
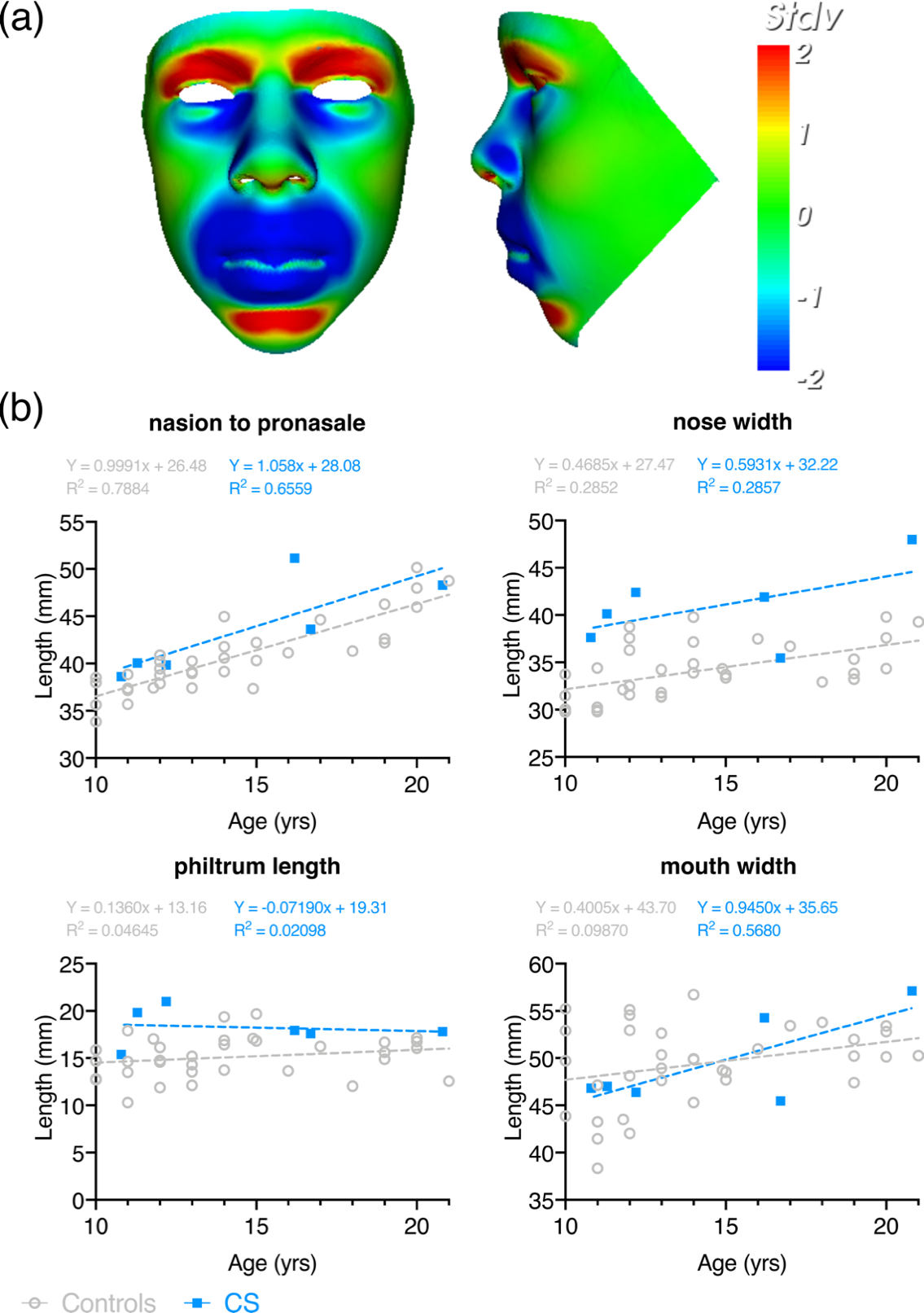
**

**Supplementary Figure 6**

**Facial abnormalities in female CS patients from 2-5 years of age. (a)** Portrait and profile views of the colour-coded comparison of female CS patients (n=3, age range: 3.1-5.5 years) with the average of the female control group (n=20, age range: 2.5-5.8 years). The colour scale of all depicted heatmaps is equal to Figure 1. **(b)** A scatter of age against different facial features is shown for a single DSM for a reduced face patch (no ears) for both age-matched controls and female CS patients. Statistically significant differences between female CS patients and age- and gender-matched controls confirm a wider nose (p=0.0101).

DSM: Dense surface model.

**
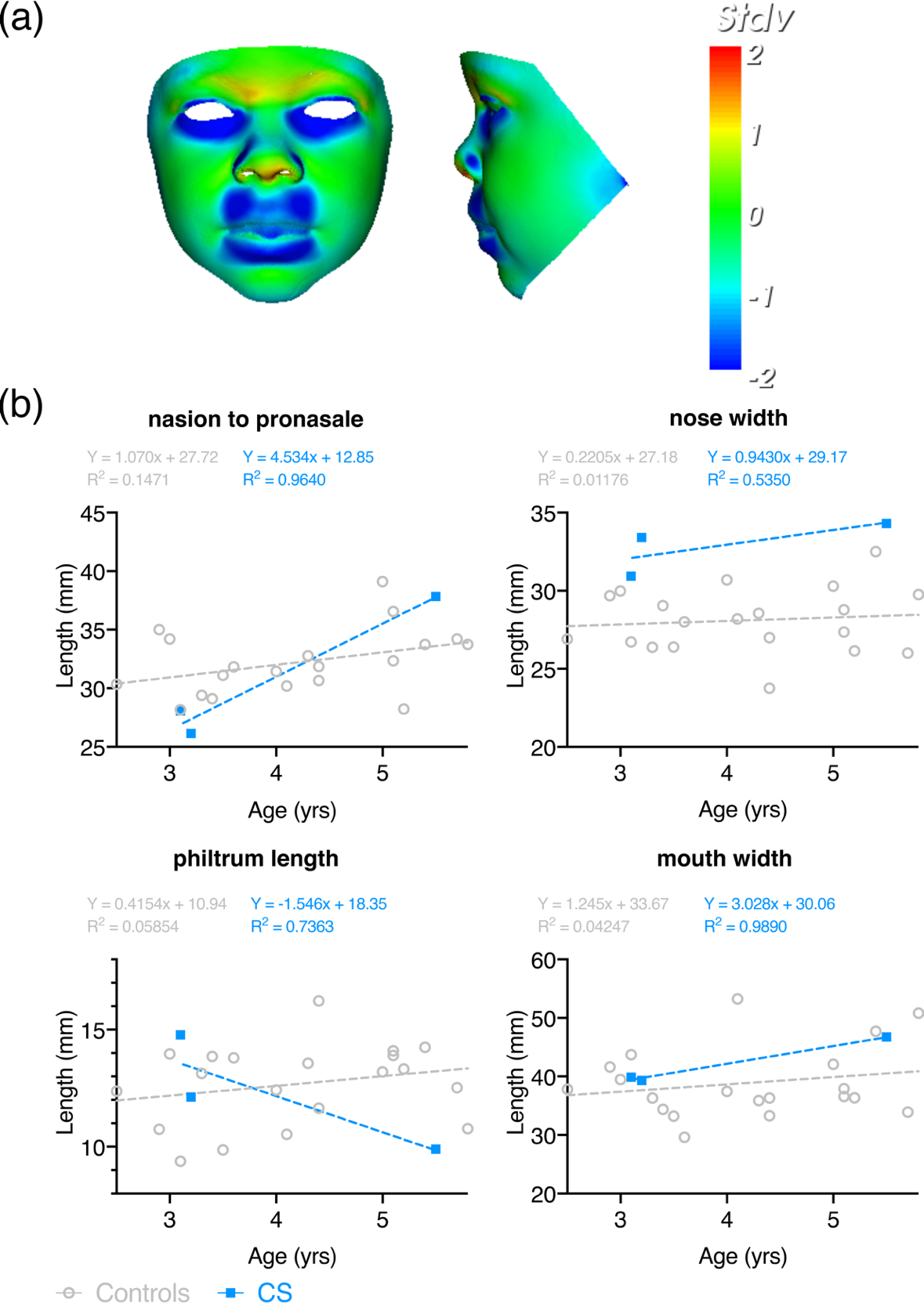
**

**Supplementary Figure 7**

**Facial abnormalities in female CS patients from 10-22 years of age. (a)** Portrait and profile views of the colour-coded comparison of female CS patients (n=7, age range: 10.0-22.0 years) with the average of the female control group (n=25, age range: 10.0-22.4 years). The colour scale of all depicted heatmaps is equal to Figure 1. **(b)** A scatter of age against different facial features is shown for a single DSM for a reduced face patch (no ears) for both age-matched controls and female CS patients. Statistically significant differences between female CS patients and age- and gender-matched controls confirm a longer philtrum (p=0.0036).

DSM: Dense surface model.

**
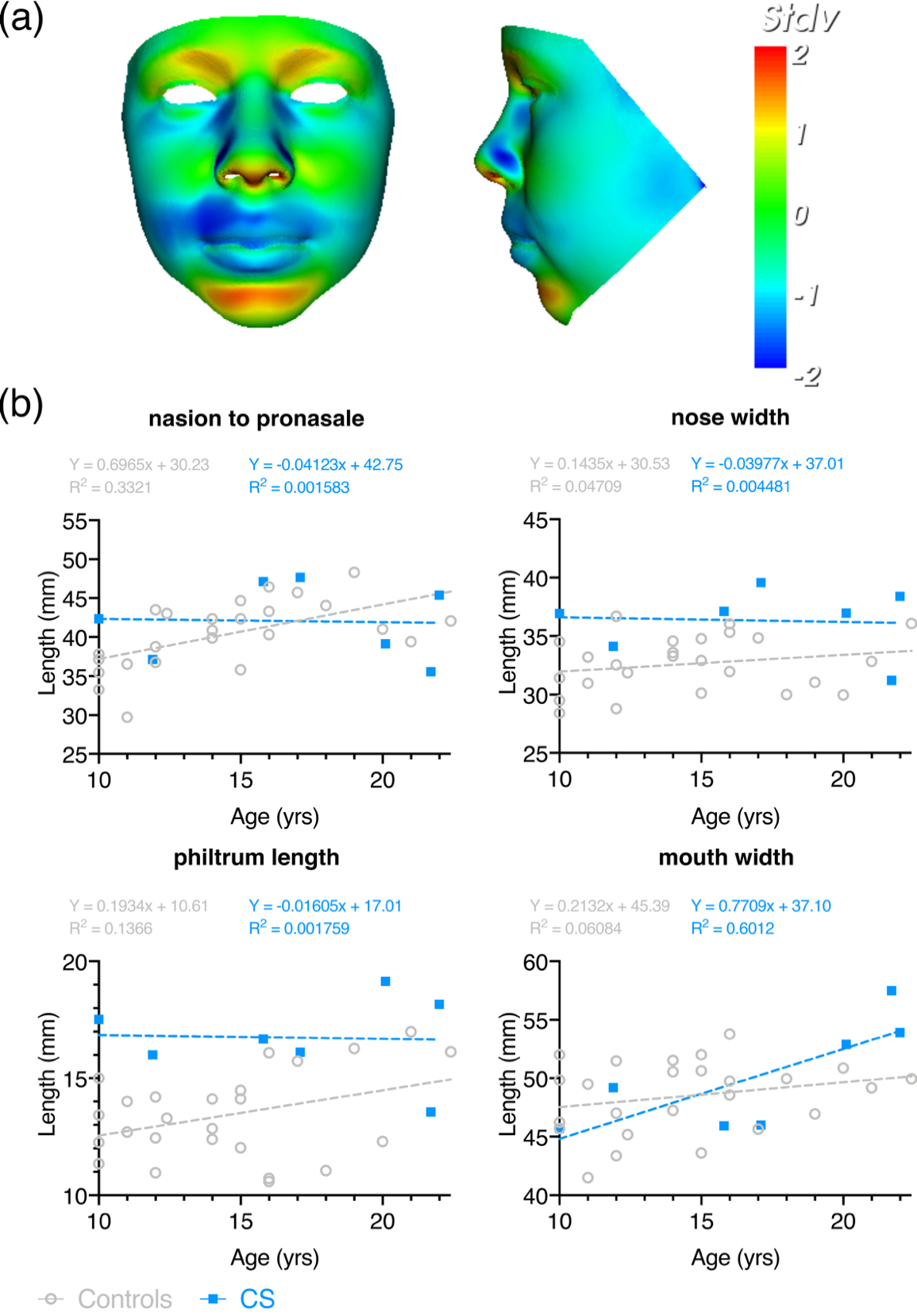
**

**Supplementary Figure 8**

**Facial growth and overall dysmorphism in age-specific CS subgroups.** (a)-(b) A scatter of age against PC1 is shown for a single DSM for a reduced face patch (no ears) for both male (a) and female (b) control and CS subgroups. The scatter is annotated by separate linear regression lines for each subgroup. PC1 reflects facial growth. Face size is significantly greater in both male CS subgroups (2-5yrs: p=0.0004; 10-22yrs: p=0.001). **(c)** A scatter of age against signature weight is shown for a single DSM for a reduced face patch (no ears) for male and female CS subgroups. Signature weight is a rough estimate of facial dysmorphism.

Sample size, male CS subgroups: n=4 (age range: 2.7-5.1 years), n=6 (10.8-20.8 years); male controls: n=17 (age range: 3.1-5.8 years), n=36 (age range: 10.0-21.0 years); female CS subgroups: n=3 (age range: 3.1-5.5 years), n=7 (age range: 10.0-22.0 years); female controls: n=20 (age range: 2.5-5.8 years), n=25 (age range: 10.0-22.4 years).

DSM: Dense surface model; PCA: principal component.


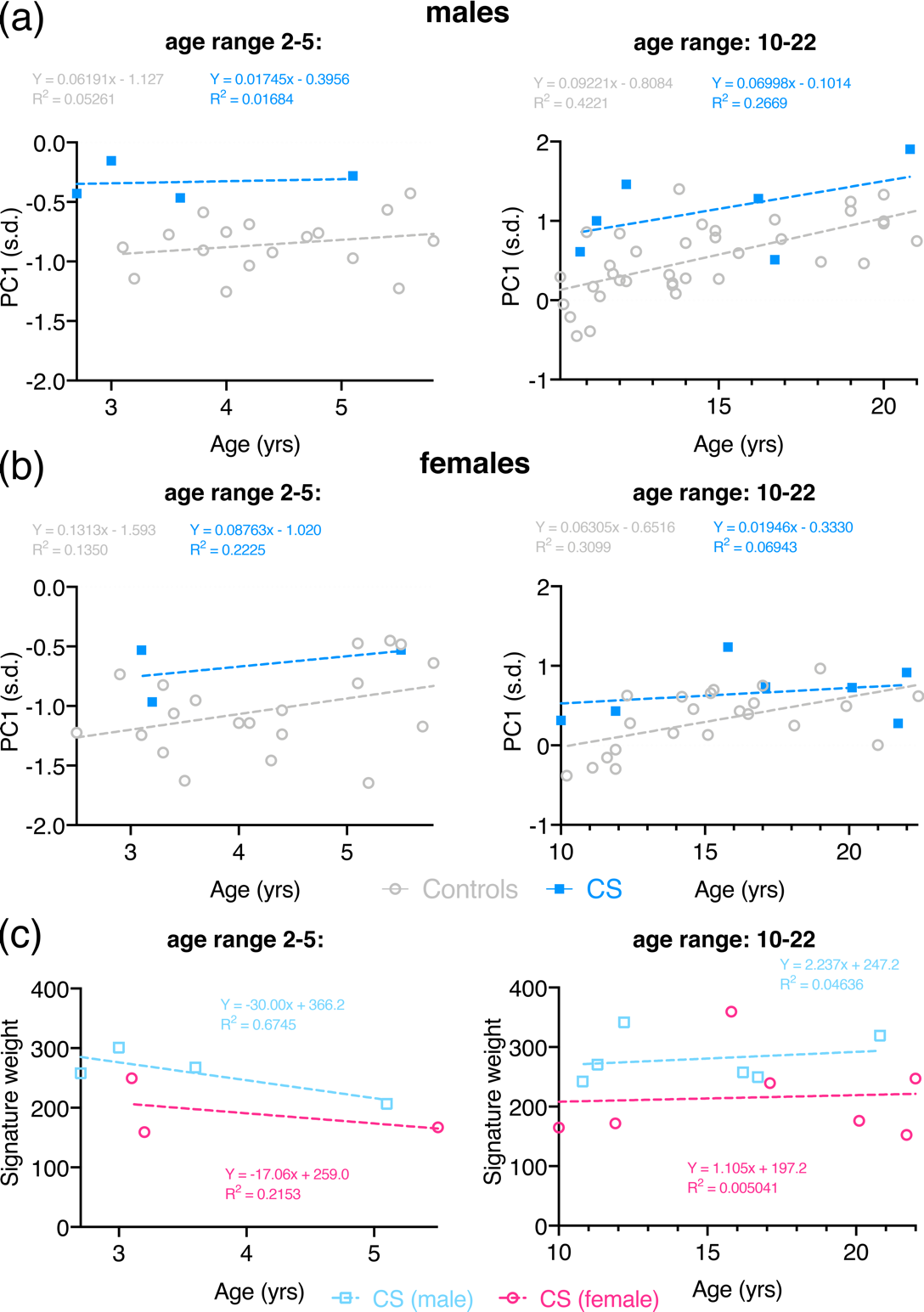


**Supplementary Figure 9**

**Facial growth in CS patients compared to other disease cohorts.**

A scatter of age against PC1 is shown for a single DSM for a reduced face patch (no ears) for CS and other genetic disease cohorts. The scatter is annotated by separate linear regression lines for each subgroup. PC1 reflects facial growth.

The difference in intercept was highly statistically significant (CS/Rasopathies and CS/RTS, p<0.0001; CS/FD, p=0.0021). When comparing CS to WD the slopes differ so much (p=0.0014), it was not possible to test whether the intercepts differ significantly. Sample size, CS, n=20 (age range: 2.7-22.0 years); Rasopathies, n=33 (age range: 2.3-21.9 years); WS, n=83 (age range: 2.1-19.7 years); RTS, n=59 (age range: 2.0-21.6 years); FD, n=28 (age range: 3.4-22.8 years).


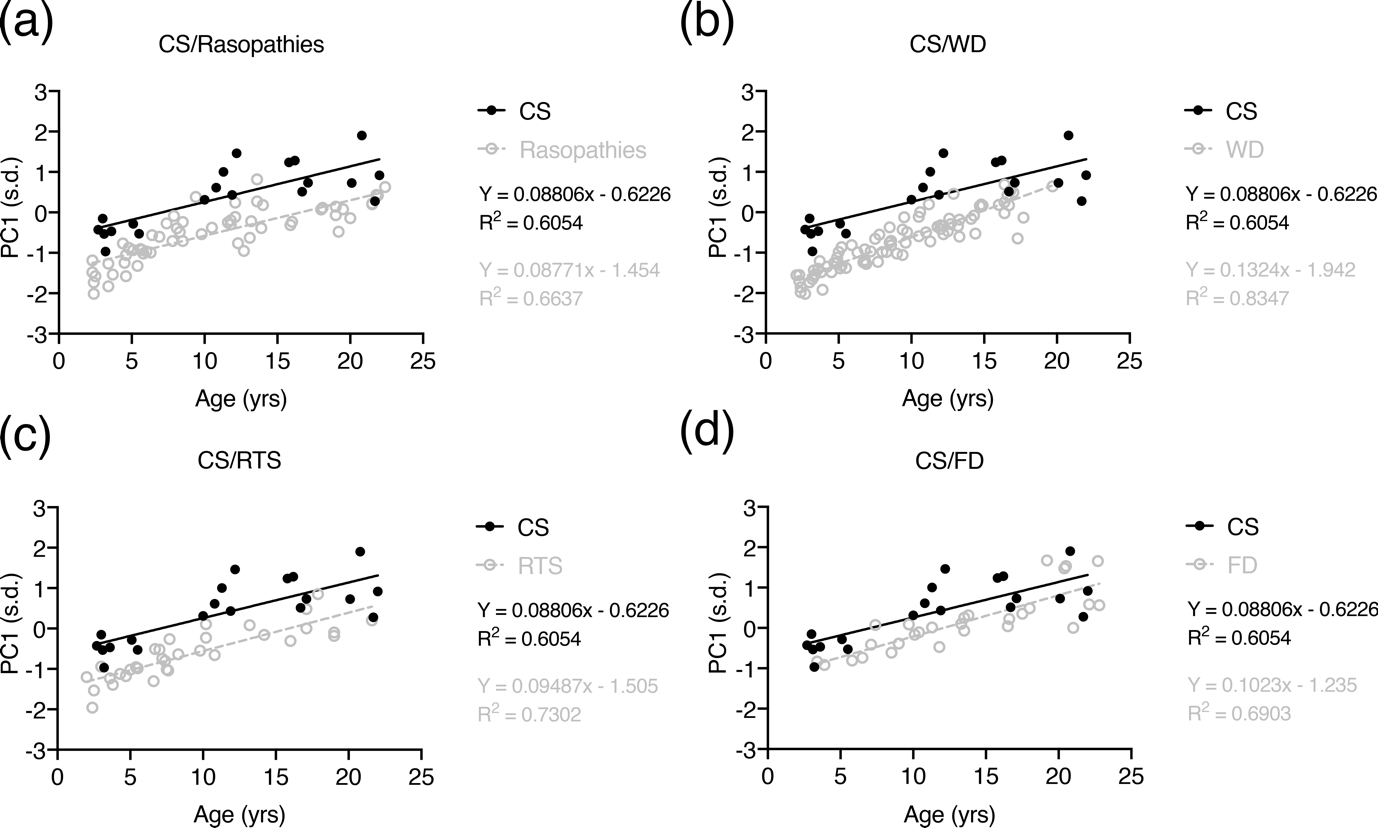


**Supplementary Figure 10**

**Facial growth in age-specific CS subgroups compared to other disease cohorts.**

A scatter of age against PC1 is shown for a single DSM for a reduced face patch (no ears) for each age-specific CS and other genetic disease subgroups. The scatter is annotated by separate linear regression lines for each subgroup. PC1 reflects facial growth.

Facial growth was statistically significantly increased for both subgroups when comparing CS/Rasopathies (both p<0.0001), CS/RTS (both p<0.0001) and CS/FD (both p<0.0001) and for the older CS/WS subgroup (p=0.0448). Sample size, CS, n=7 (age range: 2.7-5.5 years), n=13 (age range: 10.0-22.0); Rasopathies, n=18 (age range: 2.3-5.8 years), n=28 (age range: 10.5-22.4 years); WD, n=26 (age range: 2.1-5.9 years), n=35 (age range: 10.5-19.7 years), RTS, n=11 (age range: 2.0-5.5 years), n=12 (age range: 10.2-21.6 years), FD, n=3 (age range: 3.4-5.8 years), n=19 (age range: 10.1-22.8 years).


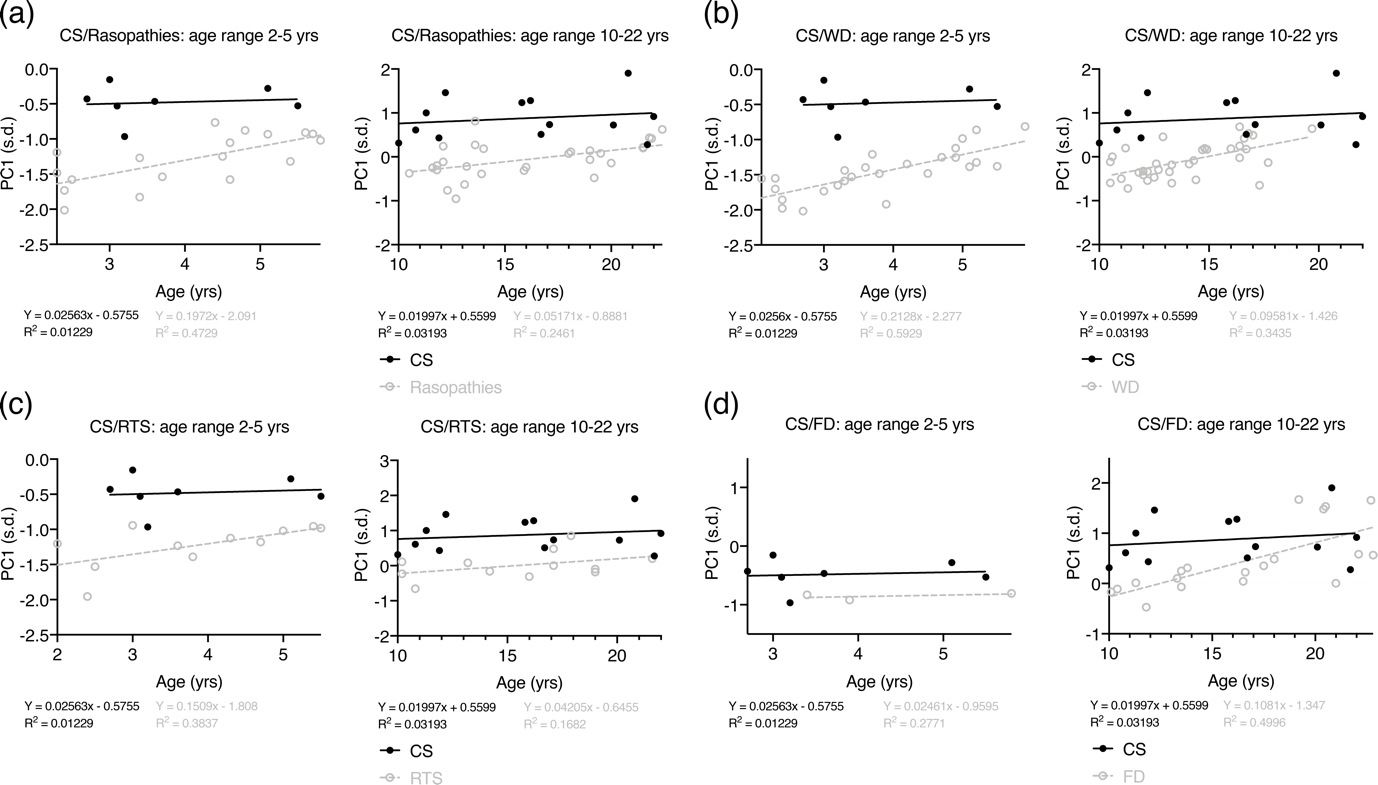


**Supplementary Figure 11**

**Overall dysmorphism in CS patients compared to other disease cohorts.**

A scatter of age against signature weight is shown for a single DSM for a reduced face patch (no ears) for male and female CS subgroups. Signature weight is a rough estimate of facial dysmorphism.

The difference in slope was highly statistically significant when comparing CS and various Rasopathies (p=0.0057). No statistical significance was found in all other cases (CS/WD, p=0.3; CS/RTS, p= 0.0862; CS/FD, p= 0.2780). Sample size, CS, n=20 (age range: 2.7-22.0 years); Rasopathies, n=33 (age range: 2.3-21.9 years); WS, n=83 (age range: 2.1-19.7 years); RTS, n=59 (age range: 2.0-21.6 years); FD, n=28 (age range: 3.4-22.8 years).


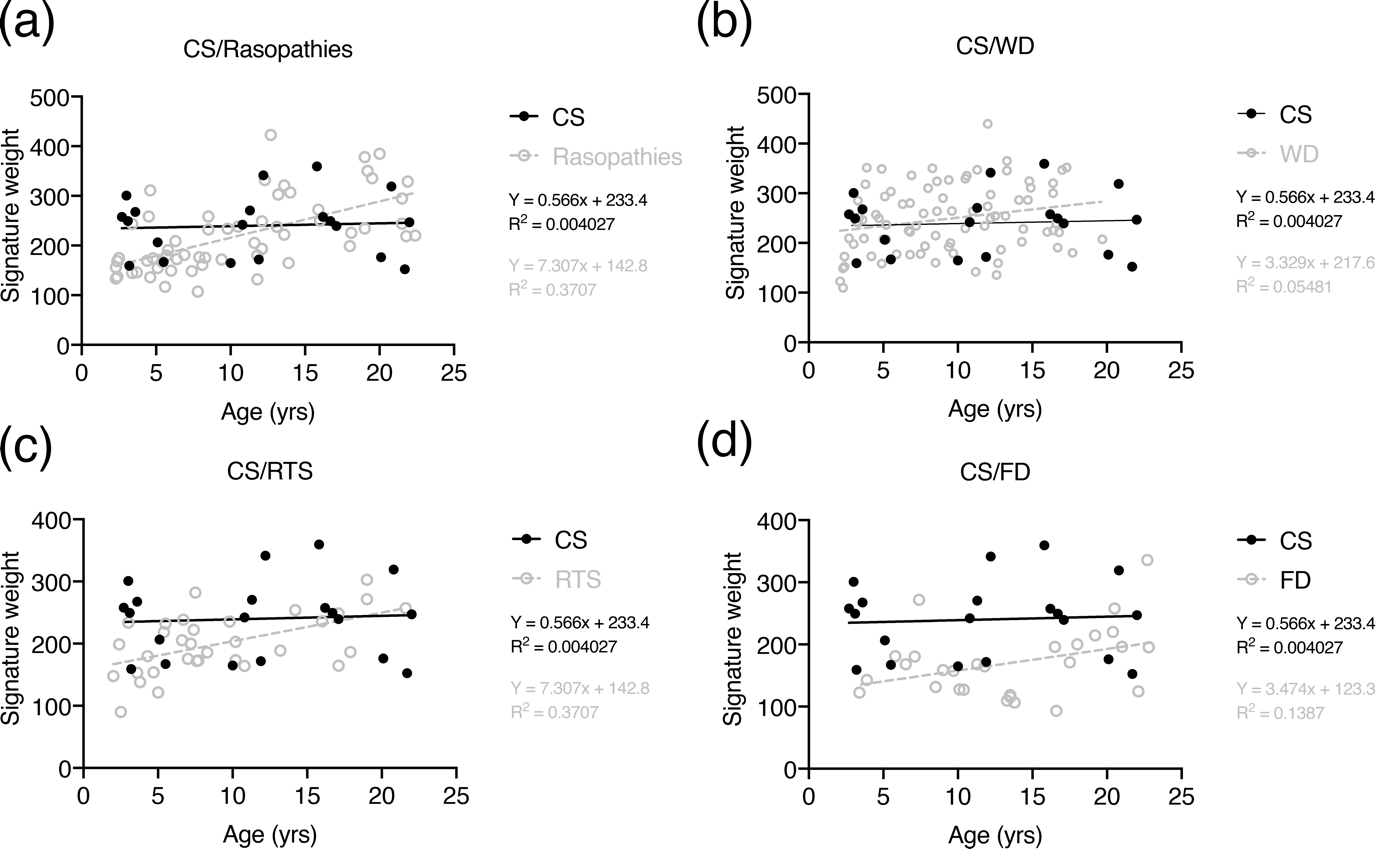


**Supplementary Figure 12**

**Overall dysmorphism in age-specific CS subgroups compared to other disease cohorts.**

A scatter of age against signature weight is shown for a single DSM for a reduced face patch (no ears) for male and female CS subgroups. Signature weight is a rough estimate of facial dysmorphism.

The difference in slope was highly statistically significant when comparing the younger subgroup of CS and WD (p=0.0353). Sample size, CS, n=7 (age range: 2.7-5.5 years), n=13 (age range: 10.0-22.0); Rasopathies, n=18 (age range: 2.3-5.8 years), n=28 (age range: 10.5-22.4 years); WD, n=26 (age range: 2.1-5.9 years), n=35 (age range: 10.5-19.7 years), RTS, n=11 (age range: 2.0-5.5 years), n=12 (age range: 10.2-21.6 years), FD, n=3 (age range: 3.4-5.8 years), n=19 (age range: 10.1-22.8 years).


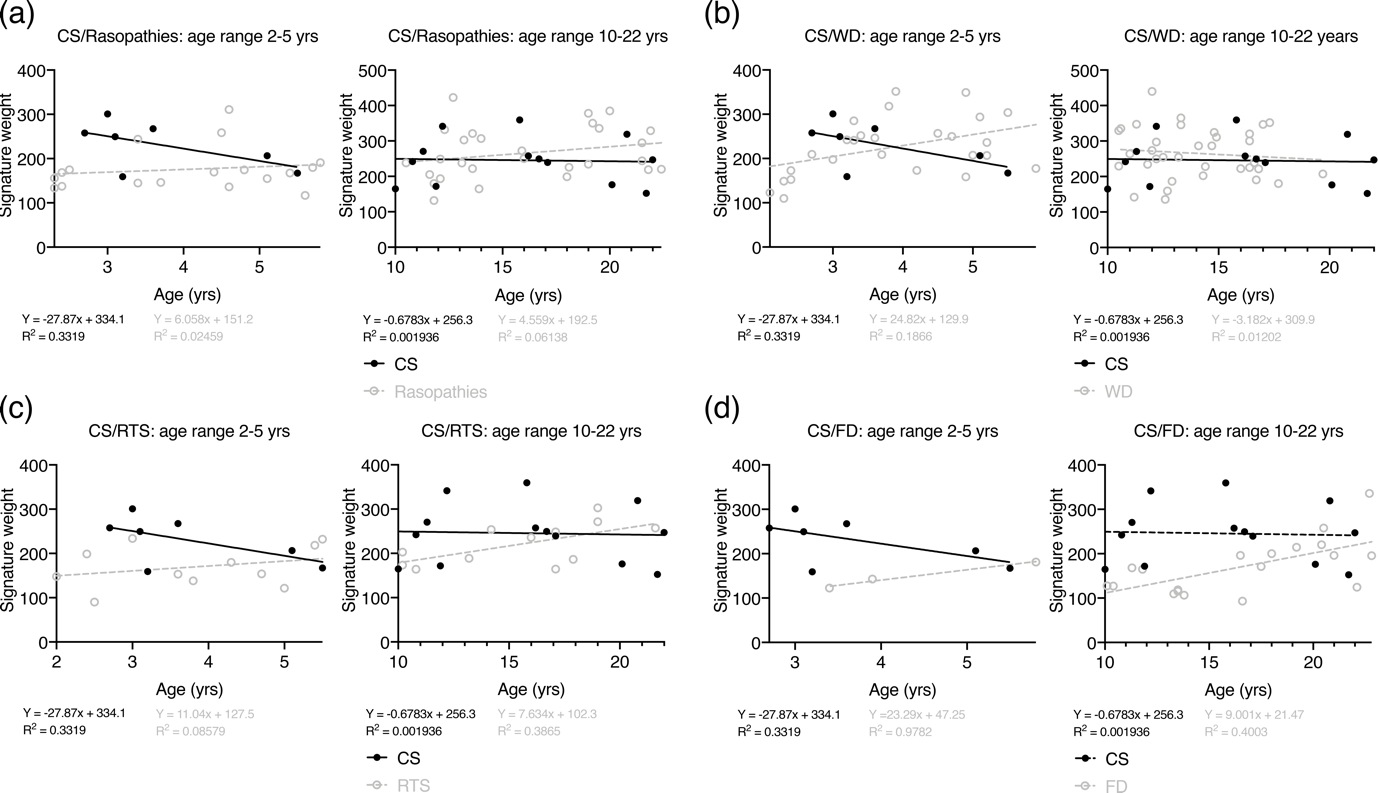


**Supplementary Table 1**

**Major Clinical Features of Cantú Syndrome found in all 20 studied subjects and compared with findings from ICSR.**

| **Patient** | **CS0001** | **CS0002** | **CS0005** | **CS0011** | **CS0013** | **CS0016** | **CS0017** | **CS0020** | **CS0024** | **CS0028** | **CS2001** | **CS2003** | **CS2004** | **CS2005** | **CS2008** | **CS2009** | **CS2010** | **CS2012** | **CS2013** | **CS2014** | **Present study** | **ICSR^a^** |
| --- | --- | --- | --- | --- | --- | --- | --- | --- | --- | --- | --- | --- | --- | --- | --- | --- | --- | --- | --- | --- | --- | --- |
| **Pregnancy/delivery** |  |  |  |  |  |  |  |  |  |  |  |  |  |  |  |  |  |  |  |  |  |  |
| Polyhydramnios | - | - | - | + | + | + | + | + | - | + | na | - | na | na | na | na | na | na | na | + | 7/12 | 34/60 |
| Gestation (weeks) | 36 | FT | FT | 29 | 34 | 35 | FT | 37 | 38 | FT | FT | 33 | 38 | FT | na | FT | FT | 32 | 37 | FT | 37.3  weeks | 33.9  weeks |
| **Skin/hair growth** |  |  |  |  |  |  |  |  |  |  |  |  |  |  |  |  |  |  |  |  |  |  |
| Cong. hypertrichosis | + | + | + | + | + | + | + | + | + | + | + | + | + | + | + | + | + | + | + | + | 20/20 | 68/72 |
| Wrinkled and/or loose skin | - | - | + | + | - | - | + | + | + | + | + | + | + | + | - | + | + | + | - | - | 13/20 | 42/69 |
| **Cardiovascular** |  |  |  |  |  |  |  |  |  |  |  |  |  |  |  |  |  |  |  |  |  |  |
| PDA | + | + | + | + | + | - | + | - | - | - | - | + | + | + | - | + | - | + | - | + | 12/20 | 44/73 |
| Aortic root dilation | + | - | - | + | + | + | + | + | + | + | na | - | - | - | na | na | - | na | na | - | 8/16 | 21/66 |
| Enlarged heart | + | + | + | + | + | + | + | + | + | + | + | + | + | + | na | + | na | + | na | - | 17/18 | 42/69 |
| Pericard. Eff. | - | + | - | + | - | + | - | + | - | - | na | + | + | - | na | na | - | - | na | - | 6/16 | 17/69 |
| Arrhythmia | - | + | - | - | - | + | - | + | + | - | na | - | + | - | na | na | - | - | na | - | 5/16 | 12/65 |
| Pulmonary hypertension | - | - | - | + | + | - | - | - | - | - | na | + | - | - | na | na | - | na | - | - | 3/16 | 16/67 |
| Blood pressure abnorm. | - | - | - | + | - | - | - | + | - | + | + | - | - | - | + | na | + | - | - | - | 6/19 | 16/71 |
| Lymphedema | - | + | + | - | - | - | - | - | - | - | - | - | - | - | na | + | - | - | - | - | 3/19 | 8/72 |
| Abnorm. blood vessels | + | + | + | + | - | + | - | + | - | - | - | - | na | - | - | na | na | na | na | - | 6/15 | 13/68 |
| Edema | - | + | + | - | + | + | + | + | - | - | + | - | + | - | na | + | + | + | + | - | 12/19 | 27/63 |
| Other | - | - | APC, MV regurg. | - | - | - | PFO | MV regurg. | Enlarged left heart, bicuspid aortic valve | - | - | - | - | MV regurg. | - | Valve does not close properly | MV regurg. | - | - | -. | 7/20 | 21/74 |
| **Neurological** |  |  |  |  |  |  |  |  |  |  |  |  |  |  |  |  |  |  |  |  |  |  |
| Headaches/migraine | M | H | M | H/M | - | - | - | M | H | - | - | - | M | - | M | M | - | - | H | - | 10/20 | 27/68 |
| Seizures | - | - | Febrile | - | + | Convulsions during sleep | - | - | Febrile | Febrile | - | - | + | - | + | - | - | - | + | - | 8/20 | 17/72 |
| Brain abnorm. | - | - | White matter lesions | Decreased white matter | External hydrocephalus | Dysgenesis of corpus callosum, diffuse leptomeningeal enhancement | - | Periventricular leukomalacia | - | - | - | - | - | - | - | Abnormally small pituitary | - | - | na | - | 6/19 | 11/49 |
| **Other** |  |  |  |  |  |  |  |  |  |  |  |  |  |  |  |  |  |  |  |  |  |  |
| Skeletal Dysplasia | - | - | + | - | + | - | - | - | - | - | - | - | na | na | - | - | na | na | na | - | 2/15 | 12/64 |
| Osteopenia/Osteoporosis | - | - | - | - | + | - | - | - | - | - | na | - | na | + | - | na | na | na | na | - | 2/14 | 4/64 |
| Scoliosis | - | + | - | - | - | - | - | + | - | - | - | - | na | + | - | - | - | - | - | - | 3/19 | 10/70 |
| Pectus carinatum | na | - | + | + | + | - | - | + | - | - | + | - | + | + | - | + | - | + | + | + | 11/19 | 17/69 |
| Joint laxity | + | + | + | + | - | - | - | + | - | - | + | - | + | + | + | + | + | + | - | + | 13/20 | 39/70 |
| Hypotonia | + | - | + | + | + | + | + | + | - | + | + | - | - | + | + | + | na | + | + | + | 15/19 | 42/65 |
| Breathing difficulties | + | + | + | + | + | + | - | + | + | + | - | + | + | + | + | - | + | + | - | + | 16/20 | 43/71 |
| Hernia | U | - | - | U/I | - | U | - | I | U | - | U | I | U | - | - | - | - | U/I | - | - | 9/20 | 25/72 |
| Developmental delay | + | - | + | + | + | + | + | + | - | + | - | + | + | - | + | + | - | + | - | + | 14/20 | 45/71 |

*Abbreviations:*

Aort. Coarct., aortic coarctation; APC, aorto‐pulmonary collateral; FT, full term; H, headache; ICSR, International Cantú syndrome registry; I, inguinal; M, migraine; MV regurg., mitral valve regurgitation; na, data not available; PFO, patent foramen ovale; U, umbilical

^a^ Numbers derive from International Cantú syndrome registry (Grange et al., 2019)
